# Chemotypes and Biomarkers of Seven Species of New Caledonian Liverworts from the Bazzanioideae Subfamily

**DOI:** 10.3390/molecules23061353

**Published:** 2018-06-05

**Authors:** Benjamin Métoyer, Nicolas Lebouvier, Edouard Hnawia, Gaëtan Herbette, Louis Thouvenot, Yoshinori Asakawa, Mohammed Nour, Phila Raharivelomanana

**Affiliations:** 1 Institut des Sciences Exactes et Appliquées (ISEA) EA 7484, Université de la Nouvelle-Calédonie, 98851 Nouméa, New Caledonia; benjamin.metoyer@gmail.com (B.M.); nicolas.lebouvier@univ-nc.nc (N.L.); edouard.hnawia@univ-nc.nc (E.H.); mohammed.nour@univ-nc.nc (M.N.); 2 Aix Marseille Univ, CNRS, Centrale Marseille, FSCM, Spectropole, Service 511, Campus Saint-Jérome, 13397 Marseille CEDEX 20, France; gaetan.herbette@univ-amu.fr; 3 Independent Researcher, 11, Rue Saint-Léon, 66000 Perpignan, France; thouloup@orange.fr; 4 Faculty of Pharmaceutical Sciences, Tokushima Bunri University, Tokushima 7708514, Japan; asakawa@ph.bunri-u.ac.jp; 5 UMR 241 EIO, Université de la Polynésie Française, 98702 Faaa, Tahiti, French Polynesia

**Keywords:** liverwort, *Bazzania*, *Acromastigum*, sesquiterpene, diterpene, bis(bibenzyl), biosynthesis, zierane, vittatin

## Abstract

Volatile components of seven species of the Bazzanioideae sub-family (Lepidoziaceae) native to New Caledonia, including three endemic species (*Bazzania marginata*, *Acromastigum caledonicum* and *A. tenax*), were analyzed by GC-FID-MS in order to index these plants to known or new chemotypes. Detected volatile constituents in studied species were constituted mainly by sesquiterpene, as well as diterpene compounds. All so-established compositions cannot successfully index some of them to known chemotypes but afforded the discovery of new chemotypes such as cuparane/fusicoccane. The major component of *B. francana* was isolated and characterized as a new zierane-type sesquiterpene called ziera-12(13),10(14)-dien-5-ol (**23**). In addition, qualitative intraspecies variations of chemical composition were very important particularly for *B. francana* which possessed three clearly defined different compositions. We report here also the first phytochemical investigation of *Acromastigum* species. Moreover, crude diethyl ether extract of *B. vitatta* afforded a new bis(bibenzyl) called vittatin (**51**), for which a putative biosynthesis was suggested.

## 1. Introduction

Liverworts are part of Bryoflora (mosses: 14,000 species, liverworts: 6000 species and hornworts: 300 species), considered as the first terrestrial plants and taxonomically indexed between algae and pteridophytes. Bryophytes possess archaic characteristics such as the absence of seeds and vascularized leaves [1]. Morphological traits such as small size of organs or relatively simple structure or high intraspecies variability and fugacity of some microscopic details (which may disappear within the plant dryness such as oil bodies), add difficulties for liverwort’s taxonomic identification. Nevertheless, many liverworts have unique organelles called oil bodies in their cells which are linked to the biosynthesis of original secondary metabolites such as mono-, sesqui- and diterpenes or phenolic compounds that could be cladistic biomarker. Most of liverwort’s sesquiterpenes are enantiomers of those found in higher plants [2].

New Caledonia is an archipelago of 18,600 km^2^ located in South Pacific region and considered as a hotspot of biodiversity [3]. In New Caledonia, 482 species and infraspecific taxa of liverworts have been described in a recent checklist [4]. The rate of endemism is comprised between 13% and 39% that makes New Caledonia as one of the richest liverwort areas in the world, together with Japan, New Zealand, and Costa Rica [5].

Bazzanioideae is a subfamily of the Lepidoziaceae family including three genera *Bazzania*, *Acromastigum* and *Mastigopelma*, this latter one had never been inventoried in New Caledonia [4]. Through the world, nearly 280 *Bazzania* species and 40 *Acromastigum* species are described [6]. In New Caledonia, 20 *Bazzania* species or varieties including 12 endemics and 16 *Acromastigum* species or varieties including 10 endemics have been described [4]. Usually, *Bazzania* species are divided into two chemotypes: albicanyl(drimenyl)-caffeate-cuparane (I) and calamenane (II) [7].

Volatile compounds of 27 specimens belonging to seven different species, including three endemics (marked with an asterisk) were investigated in order to check intra- and inter-variability of molecular contents. Studied Bazzanioideae species belong to the following genera: Acromastigum (A. tenax*, A. caledonicum*) and Bazzania (*B. parisii*, B. marginata*, *B. vittata*, *B. francana*, *B. bernieri* and *B. serrifolia*). *B. serrifolia* is a synonym of *B. bernieri* according to Kitagawa (1973) [8] but we studied here its putative specific status by separating the samples in two lots of specimens on the basis of their morphological traits.

Volatile components of diethyl ether extracts were analyzed by GC-MS-FID in order to index these plants into known *Bazzania* chemotypes. In the present work, two new molecules: an oxygenated dimer of lunularic acid (**51**) and an alcohol zierane-type sesquiterpene (**23**) are characterized for the first time (Table 1 and Table 2). They were respectively isolated from diethyl ether extract of *B. vittata* and *B. francana*. To our best knowledge, this is the first phytochemical investigation of all these species and the first chemical analysis of those belonging to the *Acromastigum* genus.

**Figure 1:**
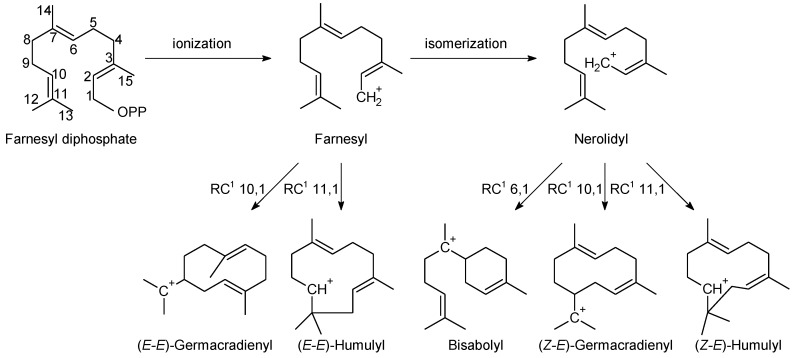
Scheme of cationic first cyclization precursor for the detected sesquiterpenes (^1^ ring closure) [19].

**Figure 2:**
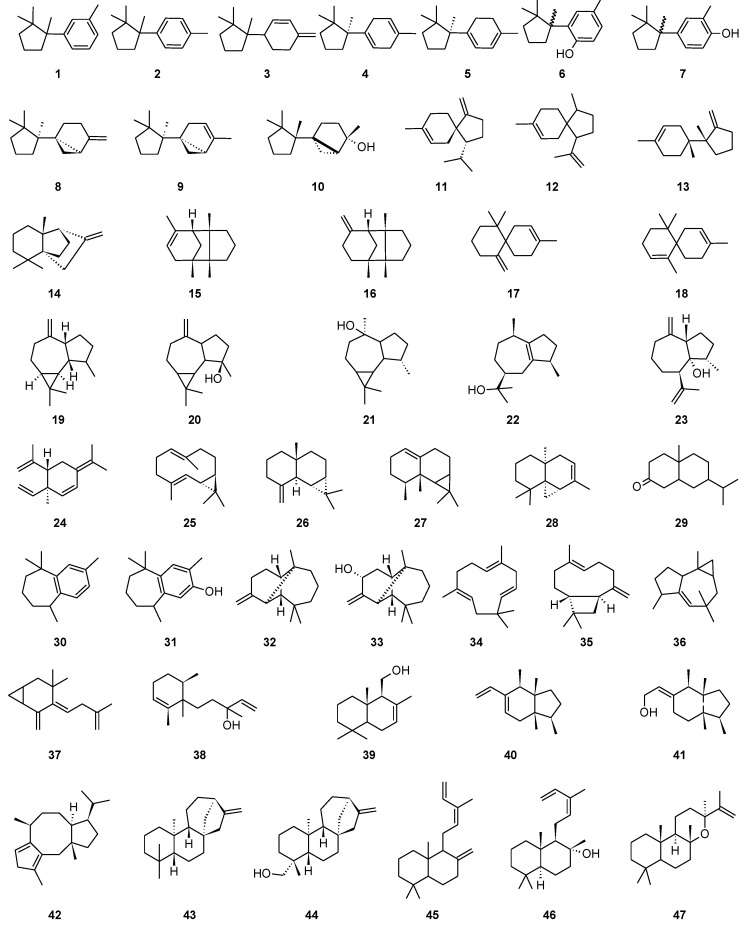
Selected compound structures detected in the studied species.

In order to sort the detected volatile compounds in this study, diterpene-type contents are listed (Table 3), listed sesquiterpene molecules were classified following their first cyclization precursors (Figure 1), and their sesquiterpene types (Table 4, Table 5 and Table 6) related to the corresponding biosynthesis pathway scheme from the literature data [9,10,11,12,13,14,15,16,17,18,19]. So, compilation of chemical composition of all studied species are gathered in Table 7. List of identified and unknown compounds is shown in Table 8, Table 9 and Table 10. Most important detected compounds for chemotaxonomy are shown in Figure 2.

## 2. Results

### 2.1. Acromastigum tenax, Bicyclogermacrane-Type

*A. tenax* is characterized by sesquiterpene components following the (*E-E*)-germacradienyl pathway (Table 4): isolepidozene (**25**) (51.5%) (previously found in *Bazzania tricrenata* [15]), elema-1,3,7(11),8-tetraene (**24**) (11.2%) and spathulenol (**20**) (10.6%) were so detected.

α-Chamigrene (**18**) (1.1%) was the only identified sesquiterpene constituent which did not belong to the (*E-E*)-germacradienyl pathway. Regarding diterpene content, only kaurane-type diterpenes were detected in *A. tenax*, namely kaur-16-en-19-ol (**44**) (7.5%) and kaur-16-ene (**43**) (6.7%). According to the literature, this is the first report of the occurrence of these two diterpene components in Bazzanioideae, although kaurane-type diterpenes had been previously found in *Bazzania*.

### 2.2. Acromastigum caledonicum, Bicyclogermacrane-Type

Three specimens of *A. caledonicum* were studied, their volatile contents were quite similar and characterized by compounds provided by the (*E-E*)-germacradienyl cation (Table 4). We detected high relative percentage values of isolepidozene (**25**) (41.0–49.0%) in the three specimens. Elema-1,3,7(11),8-tetraene (**24**) (1.4–5.8%) and 7-isopropyl-4α-methyloctahydro-2(1H)-naphthalenone (**29**) (1.2–2.9%) were also detected. The following sesquiterpenes belonging to the bisaboyl cation precursor were detected in the three specimens: β-chamigrene (**17**) (5.6–7.2%), α-chamigrene (**18**) (1.2–3.9%), acora-3,7(14)-diene (**11**) (0.8–1.4%) and 4-*epi*-α-acoradiene (**12**) (0.5–0.6%), we noticed that acorane sesquiterpenoids are known to be rare in liverworts [15]. Identified sesquiterpene belonging to the (*E-E*)-humulyl cation were respectively α-humulene (**34**) (8.0–13.4%) and β-caryophyllene (**35**) (0.6–1.7%). The only identified compound belonging to the (*Z-E*)-humulyl cation was β-longipinene (**32**) (3.4–3.8%).

### 2.3 Bazzania francana: MET062 and MET065 Zierane-Type, MET106 Microbiotane-Type and MET032 Striatane-Type

Four specimens of *B. francana* were investigated and they were found to belong to three different terpene-type compositions. Fusicocca-2,5-diene (**42**) (0.6–3.2%) was the only common constituent of these four specimens. Fusicoccane-type diterpenes are widely distributed in the genera *Plagiochila* and *Frullania* [15].

#### 2.3.1. Microbiotane-Type

Sesquiterpene content of MET106 belongs mainly to the bisaboyl cation pathway dominated by cuparane-type sesquiterpenes (widely distributed in liverworts [15]): β-microbiotene (**8**) (29.0%), α-microbiotene (**9**) (4.4%), microbiotol (**10**) (1.1%), cuparene (**2**) (1.1%), δ-cuprenene (**3**) (1.2%), α-cuprenene (**7**) (1.6%) and γ-cuprenene (**4**) (2.0%) were detected in this specimen. Other identified constituents following this pathway were mainly α-chamigrene (**18**) (9.8%) (found in several *Bazzania* species such as *B. trilobata* [20] and *B. madagassa* [21]), β-barbatene (**16**) (8.5%) (common in liverworts and encountered in the Lepidoziaceae family [15]) and myltayl-8,12-ene (**14**) (1.8%) (previously found in the Lepidoziaceae: *Kurzia trichoclados* [15] and *Bazzania japonica* [22]).

**Figure 3:**
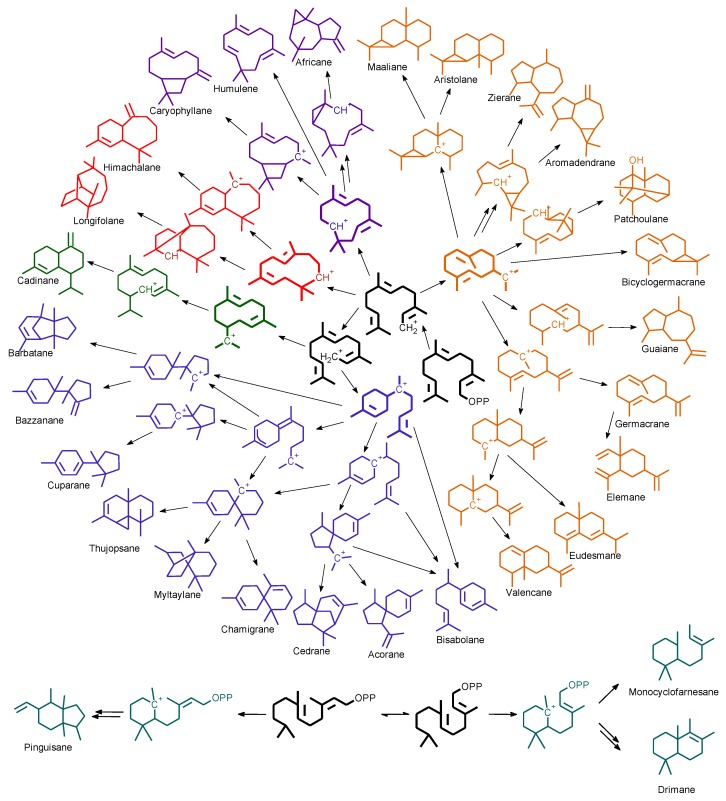
Overview of sesquiterpene types detected through the compiled biosynthesis schemes [9,10,11,12,13,14,15,16,17,18].

Compounds belonging to the (*E-E*)-germacradienyl cation precursor were also detected (Table 4): γ-maaliene (**26**) (11.9%) (detected in *Lepidozia fauriana* [23]), calarene (**27**) (5.0%) (also found in *Bazzania japonica* [22]) and viridiflorol (**21**) (1.8%), which possess different structure-skeleton but considered to be biosynthetically very close (Figure 3). (*Z*)-biformene (**45**) (8.9%), a labdane-type diterpene, was the main detected diterpene constituent.

#### 2.3.2. Striatane-Type

MET032 sample content is characterized by high percentage of striatol (**38**) (57.9%) whose structure is close to the monocyclofarnesane-type sesquiterpene structure.

Naviculol (**39**) (3.6%) (previously detected in *Bazzania novae-zelandiae* [24]), drim-7-en-11-ol (**41**) (3.6%) (=drimenol, the genera *Bazzania* and *Porella* are rich sources of drimane-type sesquiterpenes [15]), β-caryophyllene (**35**) (3.1%) and α-cuprenene (**7**) (2.8%) were detected in moderate relative percentages.

#### 2.3.3. Zierane-Type

The specimens MET062 and MET065 produced sesquiterpenes belonging to the (*E-E*)-germacradienyl cation, mainly ziera-12(13),10(14)-dien-5-ol (**23**) (86.0–90.1%) (new natural compound, structure is described below) and *allo*-aromadendrene (**19**) (1.0–6.8%).

Zierane-type sesquiterpenes are very rare in nature: zierene had been found in four different *Plagiochila* species [25] and three different zierane-type sesquiterpenes had been found in *Saccogyna viticulosa* [26], zierane-type sesquiterpene lactone had been found in *Chandonanthus hirtellus* [27]. This is the first report of zierane-type sesquiterpene regarding *Bazzania* genus.

#### 2.3.4. Structural Elucidation of Ziera-12(13),10(14)-dien-5-ol (**23**)

Compound **23** was obtained as a light-orange oil. Its molecular formula was determined to be C_15_H_26_O based on the molecular ion peak at *m*/*z* 220.1830 [M•]^+^ (calcd. for C_15_H_26_O, 220.1827) as observed in the GC/HR-EI-MS, which corresponds to four degrees of unsaturation. The IR spectrum of **23** showed absorption at 3397.5 cm^−1^ (hydroxyl), 2985.3, 2923.9, 2854.1 cm^−1^ (alkane), 1377.4 cm^−1^ (methyl), 1437.5 cm^−1^ (methylene), 3086.3, 1636.7 cm^−1^ (alkene), 1149.8 cm^−1^ (*ter*-alcohol). The ^13^C-NMR (Table 1) and HSQC spectra revealed the presence of 15 carbon resonances including three quaternary carbons, three methine, seven methylene and two methyl groups. Among the three quaternary carbons, one was an oxygenated carbon according to its chemical shift at δ_C_ 87.2 and two were *exo*-methylene carbons according to their chemical shifts at δ_C_ 151.1, 150.1. All methine groups are alkane carbons according to their chemical shifts at δ_C_ 57.6, 49.9, 46.1. Among the seven methylenes, two of them were assigned as *exo*-methylene carbons according to their chemical shifts respectively at δ_C_ 112.5 and 110.2, then so indicative of the presence of two rings.

**Figure 4:**
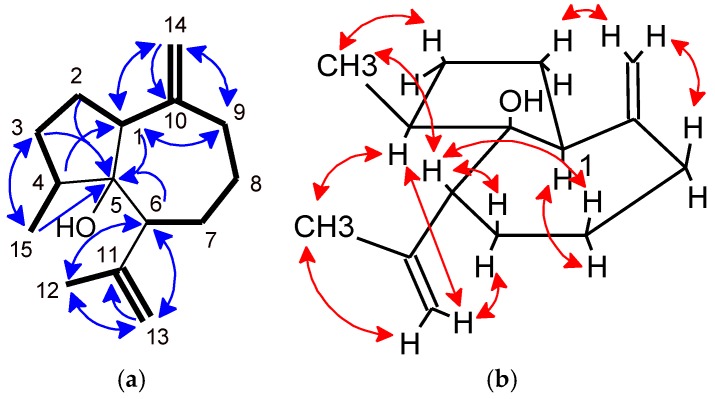
(**a**) COSY correlations (bold) and HMBC (blue arrows) key correlations of compound (**23**); (**b**) Selected NOE (red arrows) correlations of compound (**23**).

Analysis of ^1^H-NMR spectrum showed the presence of a secondary methyl at δ_H_ 0.91 (3H, d, 7.1, H-15) and two sets of *exo*-methylene groups resonating at δ_H_ 4.85 (1H, brs, H-13a), 4.78 (1H, brs, H-13b) and at δ_H_ 4.93 (1H, brs, H-14a), 4.92 (1H, brs, H-14b). The COSY spectra of **23** (Figure 4a) exhibited the presence of two spin systems, first with H-15 at δ_H_ 0.91 (d, 7.1, 3H), H-4 at δ_H_ 1.90 (m, 1H), H-3 at δ_H_ 2.16 (m, 1H) and 1.47 (m, 1H), H-2 at δ_H_ 2.01 (m, 1H) and 1.94 (m, 1H) and H-1 at δ_H_ 2.56 (t, 8.6, 1H). Then, linkages to a cyclopentane ring was deduced with HMBC correlations between H-3, H-2 and H-1 with C-5 at δ_C_ 87.2 (Figure 4a).

The second spin system with H-9 at δ_H_ 2.52 (1H, brdt, 13.2, 4.8) and δ_H_ 2.01 (1H, m), H-8 at δ_H_ 1.94 (1H, m) and δ_H_ 1.44 (1H, m), H-7 at δ_H_ 2.03 (1H, m) and δ_H_ 1.52 (1H, m) and H-6 at δ_H_ 2.30 (1H, brd, 9.8), the HMBC correlation between H-6 and C-5 at δ_C_ 87.2, and the correlations between H-14 at δ_H_ 4.93 (1H, brs, H-14a) and δ_H_ 4.92 (1H, brs, H-14b) with C-1 at δ_C_ 57.6, C-9 at 40.9, C-10 at 151.1, led to establish a cycloheptane ring. The presence of HMBC correlations between *exo*-methylene protons H-13 at δ_H_ 4.85 (1H, brs, H-13a) and δ_H_ 4.78 (1H, brs, H-13b) with C-6 at δ_C_ 49.9, C-11 at δ_C_ 150.1, C-12 at δ_C_ 23.7 (Figure 4a) afforded to put in evidence the attachment of an isopropenyl moiety at C-6.

The relative configuration was deduced by the presence of NOE correlations between H-1 at δ_H_ 2.56 and H-4 at δ_H_ 1.90, and between H-6 at δ_H_ 2.30 and methyl H-15 at δ_H_ 0.91 showing that the protons H-1 and H-4 were in the same plane, H-6 and H-15 were in the other side (Figure 4b). In comparison with precursor zierene [26,28]), the relative configuration of compound (**23**) was established as *rel*-(1*S*, 4*S*, 5*R*, 6*R*) ziera-12(13),10(14)-dien-5-ol.

### 2.4. Bazzania bernieri, Fusicoccane- and Cuparane-Type

A total of nine specimens of *B. bernieri* were investigated. Diterpene compositions were very similar. Phytane-, labdane- and fusicoccane-type diterpenes were detected co-occurring in all specimens. Main detected diterpenes were fusicocca-2,5-diene (**42**) (29.3–62.6%) and (*Z*)-biformene (**45**) (0.7–7.5%). Volatile composition of the specimen MET038 is quite different from the other specimens, and so shown apart in the Table 4, Table 5, Table 6, Table 7, Table 8, Table 9 and Table 10. Results of the major specimens of *B. bernieri* (MET028, 031, 040, 047, 063, 066, 067, 069) were pooled under the appellation BB1.

Most of detected sesquiterpenes belong to the bisaboyl cation precursor: cuparane-, myltaylane- and acorane-type sesquiterpenes were detected in all specimens, mainly δ-cuprenene (**3**) (1.7–25.2%), myltayl-8,12-ene (**14**) (0.9–3.5%) and cuparene (**2**) (1.5–3.3%).

Other sesquiterpene types belonging to the bisaboyl cation such as barbatane- (1.1–4.2%), bazzanane- (5.6–14.9%), chamigrane- (0.9–5.0%) and bisabolane-type sesquiterpenes (0.6–2.0%) were detected in all specimens except in MET038 (Table 5) while 4-*epi*-marsupellol (**33**) (2.8%) was detected only in MET038. β-Caryophyllene (**35**) (0–23.8%) was detected in several specimens.

### 2.5. Bazzania serrifolia, Fusicoccane- and Cuparane-Type

Six specimens of *B. serrifolia* were investigated. Volatile compounds content of the specimens MET092 and MET099 were quite different from the others, so these specimens were pooled apart under the appellation BS1 and the other four ones (MET041, 051-053) were pooled under the appellation BS2 as presented in Table 4, Table 5, Table 6, Table 7, Table 8, Table 9 and Table 10.

Fusicocca-2,5-diene (**42**) was detected as the major volatile component within very variable relative percentages (16.1–72.6%).

We detected in *B. serrifolia* specimens various sesquiterpene types belonging to the bisaboyl cation pathway but only cuparane-type sesquiterpene was common to all of them, mainly δ-cuprenene (**3**) (0.8–21.9%). As shown in Table 6, the specimens MET092 and MET099 were different from the others since sesquiterpenes belonging to the (*E-E*)-humulyl cation were detected, these compounds were β-caryophyllene (**35**) (29.6–38.0%), african-1-ene (**36**) (5.2–6.2%) and α-humulene (**34**) (0.0–3.5%).

### 2.6. Bazzania vitatta, Bis(bibenzyl)/Aromadendrane-Type

Two specimens of *B. vittata* were studied. Volatile compounds contents of the two specimens were similar even if 10 constituents were identified for MET060, and 18 ones for MET049. *B. vittata* was characterized by the presence of aromadendrane-type sesquiterpenes mostly by the co-occurrence in both specimens of viridiflorol (**21**) (10.8–13.5%) and guaiol (**22**) (7.4–10.0%). α-Pinguisene (**40**) (1.8–10.1%) and fusicocca-2,5-diene (**42**) (8.2–21.0%) were also detected in both *B. vittata* specimens. Although monocyclofarnesane-type sesquiterpenes are rarely detected in the Jungermanniales class [15], *B. vittata* contained high relative percentage of 4,4-dimethyl-3-(3-methylbut-3-enylidene)-2-methylene bicyclo[4.1.0] heptane (**37**) (13.9–16.0%). Observed neophytadiene (10.2–13.0%) could be an artifact from phytol degradation (moiety of esterified side chain of chlorophyll-a) during GC-FID-MS analysis [29].

Large amount of a new natural compound called “vittatin”, a dimeric form of lunularic acid (**51**) was isolated from MET049 (47% of the crude ether extract, structural identification is described below). Lunularic acid (**49a**) was detected in numerous liverworts and algae but rarely in vascular plants and was known to play a similar biological role than abscisic acid found in vascular plants such as growth inhibitory [30]. Lunularic acid (**49a**) is known to possess fungicide, algaecide and anti-hyaluronidase activities [31]. Presence of this compound in MET060 was confirmed.

Biaryl *meta*-*meta* junction observed for vittatin (**51**) is a criterion for bis(bibenzyl)s structural classification. The methylenedioxy bond observed between the two bibenzyl units of vittatin (**51**) is very rare in bis(bibenzyl)s structures [32]. Putative pathway of vittatin (**51**) is proposed below as well as the role of its hypothetic precursor in the structural biosynthesis scheme of natural bis(bibenzyl)s.

#### 2.6.1. Structural Elucidation of Vittatin (**51**)

**Figure 5:**
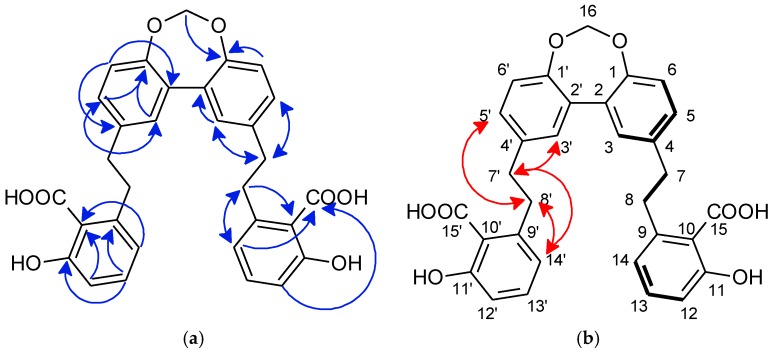
(**a**) HMBC (blue arrows) key correlations of compound (**51**); (**b**) COSY (bold) and selected NOE (red arrows) correlations of compound (**51**).

Compound (**51**) was obtained as a flaky white amorphous powder. Its molecular formula was determined to be C_31_H_26_O_8_ based on the molecular ion peak at *m*/*z* 527.1700 [M + H]^+^ (calcd. for C_31_H_27_O_8_, 527.1700) observed in the HR-ESI-MS, corresponding to nineteen degrees of unsaturation. The IR spectrum of (**51**) showed absorption at 3414.6 cm^−1^ (hydroxyl), 2946.7, and 1445.6 cm^−1^ (methylene), 1608.3, 1575.1, 1496.8 cm^−1^ (aromatic ring), 1668.6 cm^−1^ (unsaturated carbonyl), 1250.1 cm^−1^ (carboxyl), 1205.1 cm^−1^ (phenol), 1411.2, 921.7 cm^−1^ (hydroxyl). The ^13^C-NMR (Table 2) and HSQC spectra revealed the presence of only 16 carbon resonances including seven quaternary carbons, six methine and three methylene groups, suggesting a dimer form. Among the seven quaternary sp^2^ hybridized carbons, one was attributed to a carbonyl carbon according to its chemical shift at δ_C_ 170.7 and six were attributed to aromatic carbons according to their chemical shifts at δ_C_ 156.6, 153.0, 141.0, 137.8, 128.3 and 119.9. All methine groups corresponded to aromatic carbons according to their chemical shifts at δ_C_ 130.9, 128.8, 128.3, 120.6, 120.4 and 114.0. Among the three methylene groups, one was methylenedioxy carbon according to its chemical shift at δ_C_ 98.9. The ^1^H and COSY correlations of (**51**) (Figure 5b) exhibited the presence of two aromatic systems, an ABX spin system as observed in the aromatic protons at δ_H_ 7.07 (2H, d, 8.2 H-6), 7.17 (2H, dd, 8.2, 2.0, H-5) and 7.51 (2H, d, 2.0, H-3) and a second aromatic system like an AX2 spin system as observed at δ_H_ 7.20 (2H, t, 7.9, H-13), 6.75 (2H, d, 7.9, H-12) and 6.76 (2H, d, 7.9, H-14), which indicated the presence of two tri-substituted aromatic rings 1,2,4 and 1,2,3 respectively. The ^1^H and COSY spectra showed also an ethylene group at δ_H_ 2.97 (4H, m, H-8), 2.89 (4H, m, H-7) attached to C-4 and C-9 by the presence of ^3^J HMBC correlation between H-7 and C-3 at δ_C_ 128.3, C-5 at δ_C_ 128.8 and between H-8 and C-10 at δ_C_ 119.9, C-14 at δ_C_ 120.4 (Figure 5a). NOE correlations were observed between H-7 and H-3 and between H-8 and H-14 (Figure 5b). The HMBC spectrum showed two small ^4^J correlations between H-12 at δ_H_ 6.75, H-14 at δ_H_ 6.76 and C-15 at δ_C_ 170.7 (Figure 5a) which put in evidence the attachment of carboxylic group at C-10. A hydroxyl group was fixed on C-11 within a characteristic ^13^C chemical shift at δ_C_ 156.6 and a ^1^H chemical shift at δ_H_ 10.43 due to an hydrogen bond established with the the carboxylic acid group at C-15. The signal on ^1^H-NMR spectrum at δ_H_ 5.55 (2H, s, H-16) was attributed to a methylenedioxy attached on C-1 determined by the presence of ^3^J HMBC correlation between H-16 and C-1 at δ_C_ 153.3 (Figure 5a). The ^13^C- and ^1^H-NMR data of this monomer structure were similar of lunularic acid NMR data [33], the dimer form corresponded to two monomers of lunularic acid attached at C-2 by *meta-meta* junction within a methylenedioxy bridge at C-1. The structure of this new compound (**51**) was named vittatin. Spectroscopic data are available in Supplementary Materials.

#### 2.6.2. Putative Biosynthesis of Vittatin (**51**)

Bis(bibenzyl)s are biosynthesized from lunularin (**49b**) or its precursor lunularic acid (**49a**). This assumption was supported by feeding experiments using radioactive and ^13^C-labelled precursors [34]. Marchantin C synthase (isolated from a cell culture of *Marchantia polymorpha*) was supposed to be involved in the coupling mechanism of two molecules of lunularic acid (**49a**) leading to marchantin C (**48**) (type II) [35].

**Figure 6:**
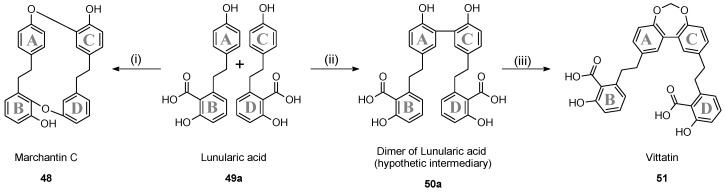
Putative biosynthesis pathway of vittatin (**51**), (i): marchantin C synthase; (ii): biaryl coupling; (iii): methylenedioxy formation.

Due to the functionalization of vittatin (**51**), hypothesis can be proposed suggesting that this bis(bibenzyl) should be formed by biaryl *meta*-*meta* coupling at the aromatic ring A (or C) of lunularic acid (**49a**) leading to a putative intermediate (**50a**). It is interesting to note that *Momordica charantia* peroxidase catalyzes biaryl *meta*-*meta* coupling with dihydroresveratrol as substrate [36]. The last step could be the formation of a methylenedioxy junction between the two phenol functions in *para* position of the aromatic cycle (A and C) leading to vittatin (**51**) as shown in Figure 6.

#### 2.6.3. Structural Relationships in Natural Bis(bibenzyl)s: Role of Putative Intermediate of Vittatin (**51**)

**Figure 7:**
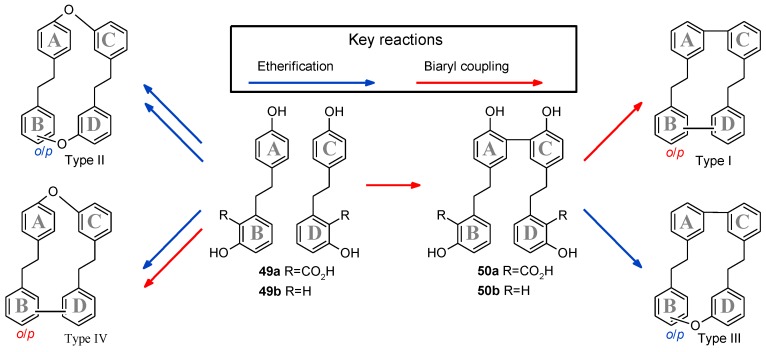
Structural relationships and interconversion of natural occuring bis(bibenzyl)s.

According to the literature, bis(bibenzyl)s are classified into four structural types (I–IV, Figure 7), each structure is composed of two bibenzyl units which differ from linkages between these units [37]. Due to its structure, the putative biosynthetic intermediate of vittatin (**50**) should be added into a previous global scheme of bis(bibenzyl) biosynthesis pathway [38] (Figure 7). This scheme highlights that the dimer of lunularin (**50b**) (=isoperrottetin A, isolated from *Radula perrottetii* [39]) and the dimer of lunularic acid (**50a**) might play the role of the precursor of the bis(bibenzyl)s of types I and III which encompassed more than 30 compounds [32].

A survey of the literature showed that bis(bibenzyl)s from liverworts of types I and III were found only in the Jungermanniopsida class. These structures were detected in liverworts from the genera *Herbertus, Lepidozia, Mastigophora, Plagiochila* and *Bazzania*, which belong to the Lophocoleineae sub-order (Jungermanniales). Nevertheless, bis(bibenzyl) compounds with a (*m*-*m*)-(C-C) bond linkage had been detected in two species which do not belong to the Lophocoleineae sub-order: *Jamesoniella colorata* (Jungermanniineae) [40] and *Radula perrottetii* (Radulineae) [39].

### 2.7. Bazzania parisii, Cuparane-Type

Main sesquiterpenes detected in *B. parisii* belong to the bisaboyl cation pathway. The barbatane- and bazzanane-type sesquiterpenes (known to share the same precursor, cf. Figure 3) were found to be dominant with β-bazzanene (**13**) (21.5%), β-barbatene (**16**) (17.8%) and α-barbatene (**15**) (1.6%). β-Chamigrene (**17**) (6.5%) and *cis*-thujopsene (**28**) (5.7%) (rare in liverworts and detected in *Bazzania trilobata* and *Lepidozia fauriana* [15]) were detected as minor compounds. Diterpene compounds were also detected as main constituents: (12*Z*)-abienol (**46**) (10.7%), 13-*epi*-manoyl oxide (**47**) (4.3%) and fusicocca-2,5-diene (**42**) (4.1%).

### 2.8. Bazzania marginata, Cuparane-Type

We detected high relative percentages of cuparane-type sesquiterpenes for *B. marginata* with two isomers of β-herbertenol (**6**) (respectively (95.9%) and (0.3%)), herbertene (1.0%) and α-herbertenol (**5**) (0.5%). Spathulenol (**20**) (1.0%), ar-himachalen-2-ol (**31**) (0.2%) and ar-himachalene (**30**) (0.2%), were detected as minor compounds.

## 3. Discussion

Important sesquiterpene-type diversity illustrated by the Figure 3 was observed among the studied samples of New-Caledonian liverworts. The genus *Bazzania* had been widely investigated in phytochemistry and most of detected sesquiterpene types belong to bazzanane-, cuparane-, barbatane-, aromadendrane-, bicyclogermacrane-, calamenane-, drimane-, chamigrane-, pinguisane-, myltaylane- and cyclomyltaylane-type [41]. Our results are consistent with the literature data except for the bicyclogermacrane-, calamenane- and drimane-type sesquiterpenes which do not seem to be widespread in *Bazzania* species from New Caledonia.

Drimane-type sesquiterpenes were detected within moderate relative percentages in one chemotype of *B. francana*. Calamenane-type, which is considered as a valuable chemotype for several Japanese *Bazzania* species [42], is the only sesquiterpene-type belonging to the (*Z*,*E*)-germacradienyl cation that had been detected in the New-Caledonian Bazzania species studied herein: calamenane-type sesquiterpene was detected with a moderate relative percentage in only one specimen of *B. serrifolia*. Thus, calamenane-type chemotype seemed to be rare in the present studied New-Caledonian *Bazzania* species: none of the analyzed specimen compositions could be chemically classified into the known chemotype II.

Isolepidozene (**25**) was detected in two specimens of *B. serrifolia* as a minor compound, but seemed to be a good biomarker for the *Acromastigum* genus which had been studied for the first time in this work. The new compound ziera-12(13),10(14)-dien-5-ol (**23**), belonging to the (*E-E*) germacradienyl cation sounds to be an important biomarker for two specimens of *B. francana* (MET062 and MET065).

**Figure 8:**
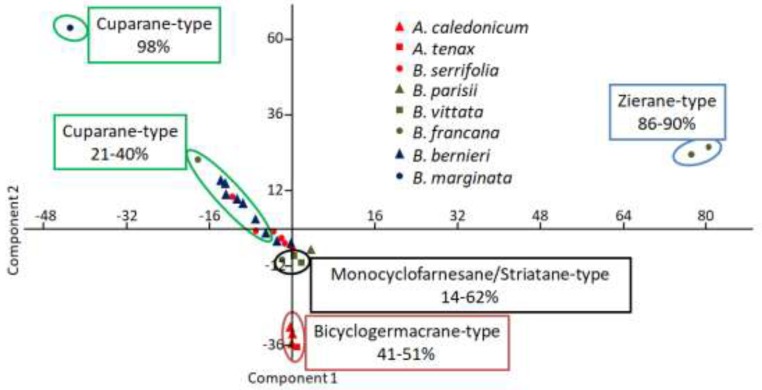
Principal Components Analysis (PCA) plot of sesquiterpene-type distribution of studied New-Caledonian Bazzanioideae order (PC1 = 36.7%; PC2 = 27.8%).

Multivariate PCA analysis of sesquiterpene type distribution (Figure 8) highlights the striatane/monocyclofarnesane-type chemotype for *B. vittata* and one specimen of *B. francana* (MET032). This fact is noteworthy since this sesquiterpene-type is very rare in the Jungermanniales order and seemed to be more specific to the Porellales order [15].

Several studied species contain high percentages of cuparane-type sesquiterpenes (namely cuparane-, herbertane- and microbiotane-type sesquiterpenes). Concerned species are the following ones: *B. marginata, B. francana* (MET106), *B. serrifolia* (MET092 and MET099) and *B. bernieri* (MET028, 063, 066, 067, 069) from which we detected from 9.3 to 97.7% of cuparane-type sesquiterpenes. This fact suggested that a “special” chemotype I based only on the cuparane-type should be more appropriate to characterize *Bazzania* species from New Caledonia. Moreover, the detection of microbiotane-type (derivative of cuparane-type) sesquiterpene for one specimen of *B. francana*, is noticeable because this compound is also very rare in liverworts [15].

We have noticed that two samples of *B. serrifolia* (MET099 and MET092) shared many common characteristics with all specimens of *B. bernieri* (except MET038) such as the high amount of β-caryophyllene (**35**) and the presence of chamigrane-, cedrane- and bisabolane-type sesquiterpenes (these four structural-type sesquiterpenes were not detected in the other specimens of *B. serrifolia* or in the specimen MET038 of *B. bernieri*). In addition, one specimen of *B. bernieri* (MET038) shared many characteristics with specimens of *B. serrifolia* (except MET099 and MET092). These data indicated proximity between *B. bernieri* and *B. serrifolia* species. Therefore, we didn’t find any evidence of chemospecific status of *B. serrifolia*, and so our phytochemical data would add more assumption of its taxonomic synonymy with *B. bernieri* [8].

Hypothesis regarding a chemotaxonomic proximity between *B. parisii* and seven *Bazzania* species from Japan (*B. bidentula*, *B. japonica*, *B. pompeana*, *B. tricrenata*, *B. tridens*, *B. trilobata* and *B. yoshinagana*) could be proposed as these species are all characterized by barbatane- and bazzanane-type sesquiterpene [42].

The liverwort *B. francana* comprised at least three chemotypes in New Caledonia: (1) striatane; (2) microbiotane; and (3) zierane chemotype. Numerous chemotypes for specimens belonging to the same species, collected in a restricted area, is a rarely observed fact but sometimes may occur, for example analysis of *Lepidozia fauriana* (Lepidoziaceae) samples collected in Taiwan, led to split them into three different chemotypes : (1) amorphane; (2) chiloscyphane; and (3) eudesmane chemotypes [15].

**Figure 9:**
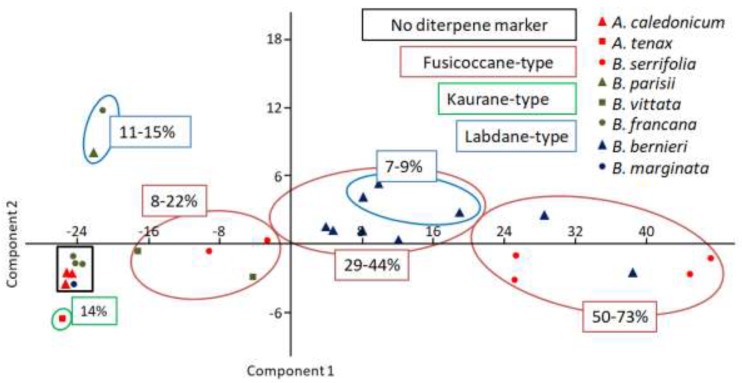
Principal Components Analysis (PCA) plot of diterpene types distribution of studied New-Caledonian Bazzanioideae species (PC1 = 96.4%; PC2 = 2.5%).

Amongst the Lepidoziaceae family, the literature data reported that fusicoccane-type diterpenes were only found in *Bazzania involuta* and *Lepidozia concinna* species [15], so our findings pointed out that fusicoccane-type diterpenes (mainly fusicocca-2,5-diene (**42**)) seemed to be specific biomarkers to *Bazzania* species of New Caledonia. As shown in PCA chart of diterpene-type distribution (Figure 9), fusicoccane-type is a characteristic biomarker of *B. serrifolia*, *B. bernieri* and *B. vittata* while labdane-type diterpene is detected mainly in *B. bernieri* and in one specimen of *B. francana* (MET106). The labdane-type diterpenes (mainly (*Z*)-biformene (**45**)) seemed to be characteristic biomarkers of *Bazzania* species from New Caledonia because this structural-type compound is very rare in the Lepidoziaceae family. Kaurane-type diterpenes were only detected in two specimens of herein studied *Acromastigum* species and may be considered as a good biomarker for *A. tenax*.

Vittatin (**51**), as a dimer of lunularic acid, could be indexed in type I and III in the bis(bibenzyl)s classification. Bis(bibenzyl)s from type I and III possess various interesting biological activities such as bactericidal towards methicillin-resistant strains like *Staphylococcus aureus* [43], antimitotic agents [44], vasorelaxant [45]. Vittatin (**51**) possesses interesting chemical functions such as carboxylic acid, phenol and a methylenedioxy moiety, these features allow numerous chemical transformation through hemisynthesis. Due to its occurrence and functionalization, vittatin (**51**) should be a valuable raw material for the synthesis of interesting bis(bibenzyl)s (type I and type III), which could have promising pharmaceutical potential.

## 4. Materials and Methods

### 4.1. General Experimental Procedure

Plant material was air-dried at room temperature and small amount of samples were crushed and extracted with Et_2_O with mortar and pestle. Extract was then purified through a Pasteur pipette packed with silica gel using Et_2_O as eluent to retrieve polar compounds. Crude extracts have been analyzed by GC-FID-MS. GC-FID-MS analysis was performed using a gas chromatograph coupled with a mass detector (Clarus^®^ 580, Perkin Elmer Inc, Waltham, MA, USA) and a flame ionization detector (Clarus^®^ 580 , Perkin Elmer Inc, Waltham, MA, USA) using helium at 1 mL/min. Capillary column was a elite-5MS (30 m × 0.25 mm, 0.25 μm) (Perkin Elmer Inc, Akron, OH, USA). Analyses were performed using EI mode. The injection temperature was set at 250 °C. Analyses were carried out using a temperature program starting from 50 °C, with an initial 3 min hold, to 250 °C with a 5 °C/min heating ramp, and keeping the final temperature stable for 15 min. Mass range was set at *m*/*z* 40–500. The individual peaks were identified by comparison of mass spectra from libraries as well as the retention indices (RI), which were calculated for all volatile constituents using a homologous series of n-alkanes C8–C32 and were compared with available literature data.

Mass Finder 2.3 library, NIST library (Gaithersburg, MD, USA), Wiley library (Hoboken, NJ, USA) were used for mass spectra comparison and identification. We used mainly NIST MS Search 2.2 software, Pherobase [46] and literature data [47] for retention index comparison to identify constituents of the crude extracts. Relative percentages of constituents were calculated with the area from the FID GC chromatogram corrected with the number of carbon of the corresponding compound (based on the MS identification).

NMR analyses were performed on a Varian (500 MHz) or Bruker AVANCE III 600 (600 MHz) NMR spectrometers (Bruker, Billerica, MA, USA). Chemical shifts are given as δ (ppm) and deuterated solvent peaks as references for ^1^H- and ^13^C-NMR spectra. Infra-Red spectra were performed using IR spectrometer (FT-IR spectrometer Frontier, Perkin Elmer Inc, Waltham, MA, USA). Optical rotation was measured with an Atago Polax D polarimeter or Anton Paar MCP200 589 nm polarimeter equipped with a sodium lamp (c in g/mL). TLC analyses were carried out on Si gel plates F_254_ (Merck, Kenilworth, NJ, USA) with cyclohexane-EtOAc (1:1 and 4:1). Detection was realized with spraying 30% aqueous H_2_SO_4_ and then heated. For normal-phase column chromatography, Si gel 60 was used (0.040–0.063, 0.2–0.5 mm, Merck).

UV analyses were measured with HPLC apparatus (Waters 2695 Separation module, Milford, MA, USA) equipped with a diode array detector (Waters 2996 photodiode array detector) on a 250 mm × 4.6 mm i.d., 5 µm, ec 250/4.6 nucleodur 100-5 C18 Ec (Macherey-Nagel). The mobile phase consisted of purified water with 0.1% formic acid (A) and acetonitrile (B) at a flow rate of 0.8 mL/min. Gradient elution was performed as follows: 0 min, 20% B; 3 min, 30% B; 11 min, 35% B; 25 min, 50% B; 37–40 min, 100% B.

HR-ESI-MS analyses were measured with a SYNAPT G2 HDMS mass spectrometer (Waters, Manchester, United Kingdom). Accurate mass measurements were performed in triplicate with two internal calibrations.

### 4.2. Plant Material

Liverwort species were identified by Mr. Louis Thouvenot. Voucher specimens were deposited at the herbarium of the Institute of Research for Development (IRD), Noumea, New Caledonia (NOU). Sample Collections were realized within scientific authorizations delivered by the South Province (N°2050-2014 and N°1234-2016) and the North Province (60912-2014). Plant material is listed in Table 11.

### 4.3. Extraction and Isolation

Plant material of *Bazzania vittata* (90 g) was extracted by maceration with Et_2_O (three times during one week). The crude extract was obtained as a green pale powder (2.09 g), was then washed through a Büchner funnel with successively: cyclohexane, dichloromethane, methanol and acetonitrile to yield vittatin (**51**) (980 mg, 47%).

Plant material of *Bazzania francana* (20.5 g) was extracted by maceration with diethyl ether (three times during one week). The obtained crude extract (610 mg) was subjected to fractionation using open silica gel column chromatography with a stepwise gradient system of cyclohexane/ethyl acetate to yield 17 fractions (FI to FXVII). Fraction II yielded to ziera-12(13),10(14)-dien-5-ol (**23**) (200 mg, 33%).

### 4.4. Compound Characterization

Ziera-12(13),10(14)-dien-5-ol (**23**): transparent light-orange oil (200 mg); [α]${}_{D}^{20}$ = −40.4 (*c* 6.92, CH_2_Cl_2_). UV (CH_3_CN/H_2_O, 3:1) λ_max_: 195, 230 nm; IR (FT-IR) ν_max_: 3397.5, 3086.3, 2985.3, 2923.9, 2854.1, 1636.7, 1437.5, 1377.4, 1149.8 cm^−1^; ^1^H-NMR and ^13^C-NMR see Table 1; HR-EI-MS: *m*/*z* 220.1830 [M•]^+^ (calcd. for C_15_H_26_O, 220.1827).

Vittatin (**51**): flaky white amorphous powder (980 mg); [α]${}_{D}^{20}$ = +200.0 (*c* 5.00, pyridine). UV (CH_3_CN/H_2_O, 3:1) λ_max_: 255.78 nm; IR (FT-IR) ν_max_: 3414.6, 2946.7, 1608.39, 1575.1, 1496.8, 1445.6, 1411.2, 1250.1, 1205.1, 921.7 cm^−1^; ^1^H-NMR and ^13^C-NMR see Table 2; HR-ESI-MS: m/z 527.1700 [M + H]^+^ (calcd. for C_31_H_27_O_8_, 527.1700)

### 4.5. Statistical Analysis

In order to investigate intra- and inter specific variability from 27 specimens of the Bazzanioideae, two data sets were included in the multivariate analysis using the software program past 3. Principal Component Analysis (PCA) was performed for variance-covariance.

First data was set up for analysis of the different sesquiterpene-types (28 sesquiterpene-types corresponding to 82 identified sesquiterpenes). Second data was set up for analysis of the different diterpene-types (four diterpene-types corresponding to 12 identified diterpenes).

## Figures and Tables

**Table 1:** NMR data of compound **23** in CDCl_3_ at 300 K (^1^H at 500 MHz, ^13^C at 125 MHz).

Compound 23			
Atom	δ_H_ (*J* in Hz)	δ_C_	HMBC *J_H→C_*
1	2.56 (t, 8.6, 1H)	57.6	5, 9, 14
2	2.01 (m, 1H)	27.1	4, 5
	1.94 (m, 1H)		
3	2.16 (m, 1H)	32.1	1, 5, 15
	1.47 (m, 1H)		
4	1.90 (m, 1H)	46.1	1, 2, 5, 15
5	^_^	87.2	
6	2.30 (brd, 9.8, 1H)	49.9	5, 7, 8, 11, 12, 13
7	2.03 (m, 1H)	31.4	6
	1.52 (m, 1H)		
8	1.94 (m, 1H)	29.8	6
	1.44 (m, 1H)		
9	2.52 (brdt, 13.2, 4.8, 1H)	40.9	1, 7, 8, 10, 14
	2.01 (m, 1H)		
10	^_^	151.1	
11	^_^	150.1	
12	1.82 (brs, 3H)	23.7	6, 11,13
13	4.85 (brs, 1H)	112.5	6, 11, 12
	4.78 (brs, 1H)		
14	4.93 (brs, 1H) 4.92 (brs, 1H)	110.2	1, 5, 9, 10
15	0.91 (d, 7.1, 3H)	16.0	3, 4, 5

s: singlet, d: doublet, t: triplet, m: multiplet, br: broad.

**Table 2:** NMR data of compound (**51**) in DMSO-d6 at 300 K (^1^H at 600 MHz, ^13^C at 150 MHz).

Compound (51)			
Atom	δ_H_ (*J* in Hz)	δ_C_	HMBC *J_H→C_*
1, 1′	-	153.0	-
2, 2′	-	128.3	-
3, 3′	7.51 (d, 2.0, 2H)	128.3	1, 2, 5, 7
4, 4′	-	137.8	-
5, 5′	7.17 (dd, 8.2, 2.0, 2H)	128.8	1, 3, 7
6, 6′	7.07 (d, 8.2, 2H)	120.6	1, 2, 4
7, 7′	2.89 (m, 4H)	36.6	3, 5, 9
8, 8′	2.97 (m, 4H)	36.2	4, 10, 14
9, 9′	-	141.0	-
10, 10′	-	119.9	-
11, 11′	-	156.6	-
12, 12′	6.75 (d, 7.9, 2H)	114.0	10, 11, 14, 15
13, 13′	7.20 (t, 7.9, 2H)	130.9	9, 11
14, 14′	6.76 (d, 7.9, 2H)	120.4	8, 10, 12, 15
15, 15′	13.27 (s, 2H)	170.7	-
16	5.55 (brs, 2H)	98.9	1
OH-11	10.43 (brs, 2H)	-	-

s: singlet, d: doublet, t: triplet, m: multiplet, br: broad.

**Table 3:**
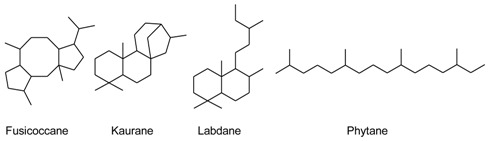
Distribution of fusicoccane-, kaurane-, labdane- and phytane-type diterpenes in studied species.

Species	Samples (METXXX)	Fusicoccane	Kaurane	Labdane	Phytane	Total
*A. caledonicum*	MET116	-	14.1	-	2.8	17.0
*A. tenax*	all	-	0.0–2.6	-	1.2–7.1	1.3–7.1
*B. bernieri*	BB1	29.3–53.7	-	3.5–7.5	0.0–1.3	34.6–60.8
	MET038	63.7	-	0.7	0.7	65.1
*B. francana*	MET032	1.6	-	1.1	0.4	3.2
	MET062,65	0.6–1.0	-	1.2–1.6	-	2.1–2.2
	MET106	3.2	-	11.0	2.2	16.4
*B. marginata*	MET048	0.3	-	-	0.3	0.6
*B. parisii*	MET109A	4.1	-	15.0	-	19.1
*B. serrifolia*	BS1	16.1–22.4	-	2.3–3.4	0.0–0.4	18.4–26.3
	BS2	50.4–72.6	-	0.8–2.1	0.0–0.3	50.4–74.7
*B. vittata*	all	8.2–21.0	-	0.0–2.2	10.2–13	20.5–34.0

**Table 4:** Distribution of detected sesquiterpene types affiliated with the (*E-E*)-germacradienyl cation (relative percentage, %).

Species	Samples (METXXX)	Maaliane	Aris.	Arom.	BG	Elemane	Germacrane	Eude.	Vala.	Guaiane	Zierane	Patc.
*A. tenax*	MET116	-	-	10.6	5.0–1.5	11.2	-	-	-	-	-	-
*A. caledonicum*	all	0.0–0.3	-	1.0–2.3	41.0–44.9	1.4–5.8	-	-	-	-	-	-
*B. bernieri*	BB1	-	-	0.4–2.3	-	0.0-0.8	-	-	-	-	-	-
	MET038	-	-	1.3	-	1.0	-	-	1.0	-	-	-
*B. francana*	MET032	-	-	1.4	-	-	-	1.0	0.6	-	-	1.5
	MET062,65	-	0.0–0.6	1.9–8.7	-	-	0.0–1.9	-	-	0.0–0.8	86.0–90.1	-
	MET106	11.9	5.0	1.8	-	0.3	-	-	5.0	-	-	-
*B. marginata*	MET048	-	-	1.0	-	-	-	-	-	-	-	-
*B. parisii*	MET109A	-	-	6.4	-	-	-	-	-	-	-	-
*B. serrifolia*	BS1	-	-	-	0.4–0.8	-	-	-	-	-	-	-
	BS2	-	-	0.0–3.6	-	-	-	-	-	-	-	-
*B. vittata*	all	-	-	12.9–13.4	-	-	-	2.9–5.5	-	7.4–10.0	-	-

Aris.: aristolane-, Arom.: aromadendrane-,BG: bicyclogermacrane-, Eude.: eudesmane-, Patc.: patchoulane- and Vala.: valancane-type.

**Table 5:** Distribution of detected sesquiterpene types affiliated with the bisaboyl cation (relative percentage, %).

Species	Samples (METXXX)	Barbatane	Bazzanane	Cuparane	Thujopsane	Myltaylane	Chamigrane	Cedrane	Acorane	Bisabolane
*A. tenax*	MET116	-	-	-	-	-	1.1	-	-	-
*A. caledonicum*	all	-	-	-	-	-	6.8–10.2	-	1.3–2.0	2.4–4.0
*B. bernieri*	BB1	1.1–4.2	5.6–14.9	3.5–28.6	-	0.9–6.3	0.9–5.0	0.0–1.5	0.0–0.6	0.6–2.0
	MET038	-	-	3.2	-	1.0	-	-	-	-
*B. francana*	MET032	0.7	-	2.9	-	0.8	-	-	-	1.0
	MET062, 65	0.0–1.6	-	-	-	-	-	-	-	-
	MET106	8.5	0.6	40.4	-	1.8	9.8	-	-	-
*B. marginata*	MET048	-	-	97.7	-	-	-		-	-
*B. parisii*	MET109A	19.4	21.4	2.0	5.7	-	6.4		-	1.4
*B. serrifolia*	BS1	0.0–1.5	2.4–3.2	9.3–23.0	-	0.0–6.3	0.9–2.0	0.2–0.5	-	0.7–0.9
	BS2	0.0–4.5	0.0–1.3	0.8–8.2	-	0.0–0.4	-	-	-	-
*B. vittata*	all	-	-	0.0–1.3	-	-	-	-	-	-

**Table 6:** Distribution of detected pinguisane-, monocyclofarnesane-, drimane-type sesquiterpenes and sesquiterpene types belonging with the (*Z-E*)-humulyl cation, (*E-E*)-humulyl cation and (*Z-E*)-germacradienyl cation precursors (relative percentage, %).

Species	Samples (METXXX)	Cation from First Cyclization Precursor								
		___________Other___________			__________(*E-E*)-Humulyl__________			(*Z-E*)-Germacradienyl	__(*Z-E*)-Humulyl__	
		Ping.	Mono.	Drimane	Africane	Humulane	Cary.	Calamenane	Hima.	Longi.
*A. tenax*	MET116	^-^	^-^	^-^	^-^	^-^	^-^	^-^	^-^	^-^
*A. caledonicum*	all	^-^	^-^	^-^	^-^	8.0–10.1	0.6–1.3	^-^	^-^	3.4–3.8
*B. bernieri*	BB1	^-^	^-^	^-^	0.0–1.5	^-^	0.0–23.8	^-^	^-^	^-^
	MET038	^-^	^-^	^-^	^-^	^-^	^-^	^-^	^-^	2.8
*B. francana*	MET032	3.6	58.8	3.6	1.1	1.0	3.1	^-^	^-^	^-^
	MET062, 65	^-^	^-^	^-^	^-^	^-^	^-^	^-^	^-^	^-^
	MET106	1.7	^-^	^-^	0.9	^-^	^-^	^-^	^-^	^-^
*B. marginata*	MET048	^-^	^-^	^-^	^-^	^-^	^-^	^-^	0.4	^-^
*B. parisii*	MET109A	^-^	^-^	^-^	^-^	^-^	^-^	^-^	1.0	^-^
*B. serrifolia*	BS1	0.3–0.4	^-^	^-^	5.4–6.5	0.0–3.5	29.6–38.0	^-^	0.0–0.7	^-^
	BS2	^-^	^-^	^-^	^-^	^-^	^-^	0.0–2.3	^-^	0.4–1.6
*B. vittata*	all	1.8–10.1	13.9–16.0	^-^	^-^	^-^	^-^	^-^	^-^	^-^

Mono.: monocyclofarnesane-, Ping.: pinguisane-, Cary.: caryophyllane-, Hima.: himachalane-, Longi.: Longifolane-type.

**Table 7:** Main constituents of studied Bazzanioideae species.

Species	Samples (METXXX)	Main Detected Compounds by GC-FID-MS		Characteristics
		Sesquiterpene	Diterpene	
*A. caledonicum*	116	isolepidozene (**25**) (41.0–49.0%) α-humulene (**34**) (8.0–13.4%)		isolepidozene (**25**)
*A. tenax*	all	isolepidozene (**25**) (51.5%) elema-1,3,7(11),8-tetraene (**24**) (11.2%)	kaur-16-en-19-ol (**44**) (7.5%) kaur-16-ene (**43**) (6.7%)	
*B .bernieri*	BB1	β-bazzanene (**13**) (5.6–14.9%) δ-cuprenene (**4**) (1.8–25.2%)	fusicocca-2,5-diene (**42**) (29.3–53.7%)	fusicoccane-type diterpene and cuparane-type sesquiterpene
	038		fusicocca-2,5-diene (**42**) (62.6%)	fusicoccane-type diterpene
*B. francana*	032	striatol (**38**) (57.9%)		striatane-type sesquiterpene
	062 065	ziera-12(13),10(14)-dien-5-ol (**23**) (86.0–90.1%)		zierane-type sesquiterpene
	106	β-microbiotene (**8**) (29.0%) γ-maaliene (**26**) (11.9%) α-chamigrene (**18**) (9.8%)	(*Z*)-biformene (**45**) (8.9%)	labdane-type diterpene and microbiotane-type sesquiterpene
*B. marginata*	048	β-herbertenol (**6**) (95.9%)		cuparane-type sesquiterpene
*B. parisii*	109A	β-bazzanene (**13**) (21.5%) β-barbatene (**16**) (17.8%)	(12*Z*)-abienol (**46**) (10.7%)	bazzanane- and barbatane-type sesquiterpene
*B. serrifolia*	BS1	β-caryophyllene (**35**) (29.6–38.0%) african-1-ene (**36**) (5.2–6.2%)	fusicocca-2,5-diene (**42**) (50.4–72.6%)	fusicoccane-type diterpene
	BS2	δ-cuprenene (**4**) (8.6–21.9%)	fusicocca-2,5-diene (**42**) (16.1–22.0%)	fusicoccane-type diterpene and cuparane-type sesquiterpene
*B. vittata*	all	4,4-dimethyl-3-(3-methylbut-3-enylidene)-2-methylenebicyclo[4.1.0]heptane (**37**) (14.0–16.0%) viridiflorol (**21**) (10.8–13.5%)	fusicocca-2,5-diene (**42**) (8.9–21.0%)	fusicoccane-type diterpene and monocyclofarnesane-type sesquiterpene

**Table 8:** Sesquiterpene composition (relative percentage, %) of the analyzed samples.

Species	RI_exp_	B. p	________B. f________			____B. s_____		____B. b____		B. m	B. v	A. t	A. c
Samples (METXXX)		109A	065, 062	106	032	BS1	BS2	BB1	038	048	all	116	all
2,4-patchouladiene	1370.2	-	-	-	1.5	-	-	-	-	-	-	-	-
anastreptene	1377.9	-	0.0–0.5	-	-	-	-	-	-	-	-	-	-
african-1-ene (**36**)	1381.8	-	-	0.9	1.1	-	5.2–6.2	0.0–1.5	-	-	-	-	-
cyclomyltaylane	1381.8	-	-	-	-	-	-	0.0–2.9	-	-	-	-	-
african-2-ene	1397.3	-	-	-	-	-	0.2–0.2	-	-	-	-	-	-
β-elemene	1397.3	-	-	0.3	-	-	-	-	-	-	-	-	-
aristol-1(2),9(10)-diene	1426.0	-	0.0–0.7	-	-	-	-	-	-	-	-	-	-
(+)-acora-3,7(14)-diene (**11**)	1426.0	-	-	-	-	-	-	-	-	-	-	-	0.8–1.4
M = 202, 91, 105(90)	1431.2	-	-	-	1.6	-	-	-	-	-	-	-	-
α-barbatene (**15**)	1432.6	1.6	-	-	-	-	-	-	-	-	-	-	-
α-microbiotene (**9**)	1434.3	-	-	4.4	-	-	-	-	-	-	-	-	-
(*E*)-caryophyllene (**35**)	1434.3	-	-	-	3.1	-	29.6–38.0	0.0–23.8	-	-	-	-	0.6–1.7
γ-maaliene (**26**)	1442.6	-	-	11.9	-	-	-	-	-	-	-	-	0.0-0.3
calarene (**27**)	1446.7	-	-	5.0	-	-	-	-	-	-	-	-	-
*cis*-thujopsene (**28**)	1450.8	5.7	-	-	-	-	-	-	-	-	-	-	-
α-chamigrene (**18**)	1450.8	-	-	9.8	-	-	0.3–0.9	0.0–1.8	-	-	-	1.1	1.2–3.9
α-pinguisene (**40**)	1459.1	-	-	1.7	-	-	0.3–0.5	-	-	-	1.8–10.1	-	-
β-barbatene (**16**)	1467.4	17.8	0.0–1.6	8.5	0.7	-	0.0–1.5	1.1–4.2	-	-	-	-	-
α-humulene (**34**)	1467.4	-	-	-	-	-	0.0–3.5	-	-	-	-	-	8.0–13.4
myltayl-8,12-ene (**14**)	1471.5	-	-	1.8	0.8	0.0–0.4	0.0–6.3	0.9–3.5	1.0	-	-	-	-
*allo*-aromadendrene (**19**)	1475.6	3.5	1.0–6.8	-	-	-	-	-	-	-	0.0–2.1	-	-
striatene	1488.0	-	-	-	0.8	-	-	-	-	-	-	-	-
4-*epi*-α-acoradiene (**12**)	1488.0	-	-	-	-	-	-	0.0–0.6	-	-	-	-	0.5–0.6
(+)-β-microbiotene (**8**)	1496.3	-	-	29.0	-	-	-	-	-	-	-	-	-
herbertene	1496.3	-	-	-	-	-	-	-	-	1.0	-	-	-
β-chamigrene (**17**)	1496.3	6.5	-	-	-	-	0.6–1.1	0.9–3.4	-	-	-	-	5.6–7.2
M = 234, 161, 203 (70)	1496.0												-
isolepidozene (**25**)	1500.4	-	-	-	-	-	0.4–0.8	-	-	-	-	51.5	41.0–49.0
M = 220, 110, 91(60)	1503.3	-	-	-	0.4	-	-	-	-	-	-	-	-
M = 204, 91, 77 (97)	1501.9	-	0.0–2.9	-		0.4–0.5	-	-	-	-	-	-	-
*cis*-γ-bisabolene	1513.5	-	-	-	-	-	-	-	-	-	-	-	1.6–2.0
isobarbatene	1513.5	-	-	-	-	0.0–0.6	-	-	-	-	-	-	-
β-bisabolene	1513.5	-	-	-	-	-	0.0–0.2	0.0–0.6	-	-	-	-	-
α-cuprenene (**7**)	1517.8	-	-	1.6	2.8	-	-	-	-	-	-	-	-
β-himachalene	1517.8	1.0	-	-	-	-	0.0–0.7	-	-	-	-	-	-
β-longipinene (**32**)	1522.2	-	-	-	-	-	-	-	-	-	-	-	3.4–3.8
cuparene (**2**)	1522.2	1.4	-	1.1	-	0.0–0.8	0.7–1.1	1.7–3.3	1.5	-	-	-	-
1,5,9-trimethyl-1,5,9-cyclododecatriene	1526.5	-	-	-	-	-	-	0.0–0.4	-	-	-	-	-
*trans*-calamenene	1530.9	-	-	-	-	0.0–0.6	-	-	-	-	-	-	-
germacrene B	1530.9	-	0.0–1.9	-	-	-	-	-	-	-	-	-	-
1,7-di-*epi*-β-cedrene	1530.9	-	-	-	-	-	0.2–0.5	0.0–1.5	-	-	-	-	-
M = 204, 93, 121 (95)	1539.0	-	-	-	-	-	-	-	-	-	-	-	0.6–0.7
β-bazzanene (**13**)	1539.6	21.5	-	0.6	-	0.0–1.3	2.4–3.2	5.6–14.9	-	-	-	-	-
ar-himachalene (**30**)	1548.3	-	-	-	-	-	-	-	-	0.2	-	-	-
γ-cuprenene (**4**)	1548.3	-	-	2.0	-	-	-	-	-	-	-	-	-
khusien-12-al	1548.3	-	-	-	0.6	-	-	-	1.0	-	-	-	-
striatol (**38**)	1561.3	-	-	-	57.9	-	-	-	-	-	-	-	-
δ-cuprenene (**3**)	1565.7	-	-	1.2	-	0.8–7.4	8.6–21.9	1.8–25.2	1.7	-	0.0–1.3	-	-
eudesma-4(15),7-dien-1β-ol	1574.3	-	-	-	1.1	-	-	-	-	-	-	-	-
M = 220, 91, 119 (90)	1575.1	-	-	-		-	0.0–1.2	-	3.1	-	-	-	-
vetiselinenol	1578.7	-	-	-	-	-	-	-	-	-	0.0–2.2	-	-
8α-hydroxy-eudesma-3,11-diene	1583.0	-	-	-	-	-	-	-	-	-	2.9–3.4	-	-
4,4-dimethyl-3-(3-methylbut-3-enylidene)-2-methylenebicyclo[4.1.0]heptane (**37**)	1591.7	-	-	-	-	-	-	-	-	-	14.0–16.0	-	-
spathulenol (**20**)	1591.7	-	0.9–1.4	-	1.0	0.0–3.6	-	0.0.4–2	0.5	1.0	-	10.6	0.8–2.3
viridiflorol (**21**)	1600.5	-	-	1.8	0.4	-	-	0.0–0.3	0.8	-	10.8–13.5	-	-
M = 220, 79, 55 (70)	1596.9	-	-	-	0.5	0.0–0.2	-	0.0–0.4	0.8	-	-	-	-
α-guaiol (**22**)	1609.5	-	0.0–0.8	-	-	-	-	-	-	-	7.4–10.0	-	0.0–0.4
M ≥ 216, 91, 135 (75)	1610.3	-	0.0–1.1	-	1.5	-	-	-	-	-	-	-	-
M = 220, 91, 105 (80)	1610.3	-	-	-	-	-	-	0.0–0.4	3.6	-	-	-	-
humulene epoxide I	1614.0	-	-	-	1.0	-	-	-	-	-	-	-	-
M = 218, 137, 95 (95)	1606.9	-	-	-	-	0.2–0.2	-	-	-	-	-	0.4	-
M = 220 105, 91 (95)	1609.8	-	-	-	-	-	0.5–1.9	-	-	-	-	-	-
M = 218, 91, 175 (85)	1620.0	-	-	0.9	0.8	-	-	-	-	-	-	-	-
M = 220, 159, 145 (80)	1621.4	-	-	-	-	-	-	-	-	-	0.0–6.6	-	-
α-bisabolene	1627.6	-	-	-	1.0	-	-	0.0–0.7	-	-	-	-	0.3–2.5
M = 232, 145, 91 (90)	1633.0	-	-	-	0.5	-	-	-	-	-	-	-	-
M = 218, 161, 91 (60)	1640.2	-	-	-	0.4	-	-	-	-	-	-	-	-
M = ?, 105, 77 (75)	1634.9	-	-	-		-	0–0.4	-	-	-	0.0–6.0	4.5	0.0–1.0
microbiotol (**10**)	1645.7	-	-	1.1	-	-	-	-	-	-	-	-	-
M = 218, 91, 79 (65)	1648.0	-	-	-	-	-	-	-	-	-	-	-	0.4–1.1
gymnomitr-3(15)-ene-4α-ol	1651.5	-	-	-	-	0.0–3.9	-	-	-	-	-	-	-
M = 234, 91, 107 (95)	1648.9	-	-	-	0.9	-	-	-	-	-	-	-	-
ziera-12(13),10(14)-dien-5-ol (**23**)	1659.3	-	86.0–90.1	-	-	-	-	-	-	-	-	-	-
M = 218, 91, 145 (85)	1662.4	-	-	-	1.6	-	-		-	-	-	-	-
M = 234, 91, 145 (95)	1679.3	-	-	0.3	0.5	-	-	-	-	-	-	-	-
M = ?, 93, 67 (50)	1682.7	-	-	-	-	-	-	-	-	-	-	-	1.4–4.6
M = 236, 112, 91 (80)	1683.7	-	-	-	0.6	-	0.0–1.1	-	1.6	-	-	-	-
α-bisabolol	1691.0	-	-	-	-	-	0.7–0.7	0.4–1.1	-	-	-	-	-
(+)-β-herbertenol (**6**)	1709.6	-	-	-	-	-	-	-	-	0.3	-	-	-
7-isopropyl-4α-methyloctahydro-2(1H)-naphthalenone (**29**)	1714.4	-	-	-	-	-	-	-	-	-	-	-	1.2–2.9
M = 234, 91, 105 (45)	1711.4	-	-	-	1.4	-	-	-	-	-	-	-	-
5-hydroxycalamenene	1728.8	-	-	-	-	0.0–1.7	-	-	-	-	-	-	-
M = 234, 91, 159 (70)	1737.0	-	-	-	2.5	-	-	-	-	-	-	-	-
α-herbertenol (**5**)	1748.1	0.7	-	-	-	-	-	-	-	0.5	-	-	-
4-*epi*-marsupellol (**33**)	1748.1	-	-	-	-	0.4–1.6	-	-	2.8	-	-	-	-
naviculol (**39**)	1752.9	-	-	-	3.6	-	-	-	-	-	-	-	-
elema-1,3,7(11),8-tetraene (**24**)	1752.9	-	-	-	-	-	-	0.0–0.8	1.0	-	-	11.2	1.4–5.8
M = 234, 161, 219 (75)	1764.2	-	-	-	-	-	-	-	-	-	0.0–12.0	-	-
γ-curcumen-15-al	1770.9	1.4	-	-	-	-	-	-	-	-	-	-	-
(−)-β-herbertenol	1781.7	-	-	-	-	-	-	-	-	95.9	-	-	-
drimenol (**41**)	1786.5	-	-	-	3.6	-	-	-	-	-	-	-	-
M = 218, 107, 91(45)	1788.3	-	-	-	-	-	1.5–3.4	0.0–0.9	4.5	-	-	-	-
M = 220, 91, 105 (70)	1796.5	-	-	-	-	-	0.0–1.1	-	-	-	-	-	-
ar-himachalen-2-ol (**31**)	1821.0	-	-	-	-	-	-	-	-	0.2	-	-	-
M ≥ 236, 69, 95(90)	1821.5	-	-	-	-	-	-	0.0–1.2	-	-	-	-	0.6–1
M = ?, 91, 95 (70)	1820.9	-	-	-	-	-	0.8–1.4	-	-	-	-	-	-
M ≥ 234, 145, 91 (95)	1831.1	-	-	-	0.8	-	-	-	-	-	-	-	-
M = 248, 91, 105 (95)	1926.1	-	-	-	-	-	-	-	-	-	-	-	4.7–7.8
M = 248, 163, 91 (70)	1935.5	-	-	-	-	-	-	-	-	-	-	-	0.6–1.3
M = 236, 69, 55 (85)	1990.3	-	-	-	-	-	0.0–1.3	-	-	-	-	-	-
**determined sesquiterpene**		**61.0**	**93.9–97.8**	**82.4**	**81.0**	**2.2–10.3**	**62.6–73.9**	**32.3–60.0**	**11.4**	**99.1**	**47.2–48.1**	**74.4**	**74.6–87.0**
**undetermined sesquiterpene**		**2.5**	**0.0–3.9**	**1.2**	**14.0**	**3.5–8.9**	**0.8–0.8**	**0.3–1.4**	**13.6**	**-**	**12.0–12.6**	**4.9**	**10.8–16.4**

B. p: *Bazzania parisii*, B. f: *B. francana*, B. s: *B. serrifolia*, B. b: *B. bernieri*, B. m: *B. marginata*, B. v: *B. vittata*, A. t: *Acromastigum tenax*, A. c: *A. caledonicum*.

**Table 9:** Diterpene composition (relative percentage, %) of the analyzed samples.

Species	RI_exp_	B. p	________B. f________			____B. s_____		____B. b____		B. m	B. v	A. t	A. c
Sample (METXXX)		109A	065, 062	106	032	BS1	BS2	BB1	038	048	all	116	all
M = 272, 121, 229 (70)	1802.0	-	-	-	-	0.4–0.5	1.1–1.7		2.5	-	-	-	-
neophytadiene I	1831.0	-	-	2.2	0.4	0.0–0.3	0.0–0.4	0.0–0.6	0.7	0.3	3.4–13	0.7	0.3–1.8
neophytadiene II	1841.0	-	-	-	-	-	-	0.3–0.9	-	-	0.0–6.8	1.8	0.9–5.3
M ≥ 272,135, 122 (45)	1852.4	0.2	-	-	-	0.5–0.5	-		-	-	-	-	-
neophytadiene III	1866.0	-	-	-	-	-	-	-	-	-	-	0.3	-
M = 272, 135, 91 (80)	1881.7	-	-	-	-	0.0–0.5	-		-	-	-	-	-
labda-7,13,14-triene	1891.0	-	-	-	-	0.0–1.0	0.0–0.7	-	-	-	-	-	-
M ≥ 281, 95, 107 (70)	1976.9	-	-	-	-	-	0.0–0.3		-	-	-	-	-
(*Z*)-biformene iso1 (**45**)	2000.5	-	0.0–0.4	8.9	0.4	0.0–1.7	2.3–2.8	3.5–7.5	0.7	-	0.0–2.2	-	-
M = 272, 73, 91 (15)	2009.1	-	-	-	-	5.9–6.8	-		-	-	0.0–2.3	-	-
13-*epi*-manoyl oxide (**47**)	2016.9	4.3	0.8–1.2	-	0.5	-	-	-	-	-	-	-	-
fusicocca-2,5-diene (**42**)	2022.4	4.1	0.6–1.0	3.2	1.6	50.4–72.6	16.1–22.3	29.3–53.7	62.6	0.3	8.2–21.0	-	-
(*Z*)-biformene iso 2	2027.9	-	0.0–0.4	2.1	-	-	-	-	-	-	-	-	-
manoyl oxide	2038.8	-	-	-	0.3	-	-	-	-	-	-	-	-
M ≥ 288, 179, 81 (70)	2044.6	0.4	-		-	-	-		-	-	-	-	-
fusicocca-3,5-diene	2060.7	-	-		-	-	0.0–0.2	0.0–0.4	1.1	-	-	-	-
M ≥ 272, 95, 81 (45)	2066.8	-	-		-	-	0.0–3.2		-	-	-	-	-
(−)-kaur-16-ene (**43**)	2071.6	-	-		-	-	-	-	-	-	-	6.7	0.0–1.2
M ≥ 270, 69, 105 (80)	2103.1	-	-		-	-	-		0.9	-	-	-	-
M> = 278, 71, 95 (85)	2110.5	-	-	-	0.7	0.0–0.5	0.0–1.1		0.6	-	0.0–8.9	0.8	0.3–0.8
(12*Z*)-abienol (**46**)	2196.6	10.7	-	-	-	-	-	-	-	-	-	-	-
M ≥ 270, 105, 119 (90)	2212.0	-	-	-	-	-	0.0–2.6	-	-	-	-	-	-
M = 288, 95, 107(80)	2256.5	-	-	-	-	-	0.0–1.6	-	-	-	-	-	-
M ≥ 286, 95, 107 (90)	2294.0	-	-	-	-	-	0.0–4.3	-	-	-	-	-	-
M ≥ 341, 95, 147 (90)	2294.6	1.1	-	-	-	-	-	-	-	-	-	-	-
M ≥ 286, 79, 91 (95)	2310.2	-	-	-	-	-	0.0–5.1	-	-	-	-	-	-
M ≥ 286, 95, 119 (90)	2321.8	-	-	-	-	-	0.0–8.0	-	-	-	-	-	-
M = 286, 81, 95 (85)	2325.3	-	-	-	-	0.0-0.3	-	-	-	-	-	-	0.0–0.6
M ≥ 286, 95, 243 (80)	2339.9	-	-	-	-	-	0.0–3.5	-	-	-	-	2.5	0.0–0.2
M ≥ 355, 83, 286 (90)	2353.9	1.1	-	-	-	-	-	-	-	-	-	-	-
M ≥ 286, 91, 243 (85)	2355.6	-	-	-	-	-	0.0–2.0	-	-	-	-	-	-
kaur-16-en-19-ol (**44**)	2390.7	-	-	-	-	-	-	-	-	-	-	7.5	0.0–1.5
M ≥ 288, 137, 19 (55)	2402.9	-	-	-	-	0.0–0.6	3.8–5.5	-	-	-	-	-	-
M ≥ 286, 121, 79 (85)	2405.3	12.9	-	-	-	-	-	-	-	-	-	-	-
M ≥ 286,95, 107 (50)	2408.7	-	-	-	-	0.0–0.5	-	-	-	-	-	-	-
M ≥ 286, 55, 95 (95)	2413.5	-	-	-	-	-	1.7–3.9	-	-	-	-	-	-
M ≥ 286,137, 95 (80)	2487.2	-	-	-	-	-	1.4–6.7	-	-	-	-	-	-
**determined diterpene**		**19.1**	**2.1–2.2**	**16.4**	**3.2**	**50.4–74.7**	**18.4–26.3**	**34.6–60.8**	**65.1**	**0.6**	**20.5–34**	**17.0**	**1.3–7.1**
**undetermined diterpene**		**15.5**	**-**	**-**	**0.7**	**11.1–29.7**	**6.9–10.4**	**2.7–7.1**	**4.0**	**-**	**0.0–11.1**	**3.2**	**0.3–1.2**

B. p: *Bazzania parisii*, B. f: *B. francana*, B. s: *B. serrifolia*, B. b: *B. bernieri*, B. m: *B. marginata*, B. v: *B. vittata*, A. t: *Acromastigum tenax*, A. c: *A. caledonicum*.

**Table 10:** Non terpenic constituent composition (relative percentage, %) of the analyzed samples.

Species	RI_exp_	B. p	________B. f________			____B. s_____		____B. b____		B. m	B. v	A. t	A. c
Sample (METXXX)		109A	065, 062	106	032	BS1	BS2	BB1	038	048	all	116	all
alkane	1492.1	-	-	-	-	-	-	-	-	-	0.0–0.4	-	-
ethyl *p*-ethoxybenzoate	1526.5	0.5	-	-	-	-	-	-	-	-	3.5–6.7	0.5	-
1-(2-benzyloxyethyl) cyclohexene	1605.0	-	-	-	-	0.0–1.7	-	0.0–0.8	2.9	-	-	-	-
1,5-diphenyl-1,4-pentadien-3-one	1641.2	0.8	-	-	-	-	-	-	-	-	-	-	-
alkane	1651.5	-	-	-	1.1	-	-	-	-	0.1	-	-	-
methyl 4,7-octadecadiynoate	1686.4	-	-	-	-	-	-	-	-	-	-	-	0.7–1.5
(2-methylene-cyclohexyl)-phenyl-methanol	1767.3	-	-	-	-	0.0–1.8	-	0.0–0.7	3.1	-	-	-	-
aliphatic alcohol	1995.3	-	-	-	-	-	-	-	-	0.2	-	-	-
aliphatic alcohol	2082.5	0.6	-	-	-	-	-	-	-	-	0.0–3.8	-	-
**total**		**2.0**	**-**	**-**	**1.1**	**0.6–3.4**	**-**	**0.3–1.5**	**6.0**	**0.3**	**6.7–7.7**	**0.5**	**0.7–1.5**

B. p: *Bazzania parisii*, B. f: *B. francana*, B. s: *B. serrifolia*, B. b: *B. bernieri*, B. m: *B. marginata*, B. v: *B. vittata*, A. t: *Acromastigum tenax*, A. c: *A. caledonicum*.

**Table 11:** Sample collection geodata informations.

Species	Voucher Specimen	Date of Collection	Ecosystem	GPS		Collection Site
				South	East	
A. caledonicum*	MET113B	06-2016	maquis	22.27	166.95	Plateau de Goro
	MET107	06-2016	maquis	22.27	166.97	Plateau de Goro
	MET109B	06-2016	maquis	22.27	166.95	Plateau de Goro
A. tenax*	MET116	06-2016	maquis	22.28	166.96	Plateau de Goro
*B. bernieri*	MET038	11-2014	maquis	21.91	166.34	Tontouta
	MET028	08-2014	rain forest	22.18	166.50	Koghis
	MET031	08-2014	rain forest	22.18	166.51	Koghis
	MET040	11-2014	maquis	21.91	166.34	Tontouta
	MET047	11-2014	rain forest	21.62	165.88	Dogny
	MET063	06-2015	rain forest	22.22	166.66	Mouirange
	MET066	06-2015	rain forest	22.17	166.79	Marais Kiki
	MET067	06-2015	rain forest	22.17	166.79	Marais Kiki
	MET069	06-2015	rain forest	22.17	166.79	Marais Kiki
*B. francana*	MET062	06-2015	rain forest	22.22	166.66	Mouirange
	MET065	06-2015	rain forest	22.17	166.79	Marais Kiki
	MET032	08-2014	rain forest	22.18	166.5	Koghis
	MET106	06-2016	maquis	22.28	166.97	Goro
B. marginata*	MET048	11-2014	rain forest	21.62	165.88	Dogny
*B. parisii*	MET109A	06-2016	rain forest	22.27	166.95	Dogny
*B. serrifolia*	MET041	09-2014	maquis	21.91	166.34	Tontouta
	MET051	11-2014	rain forest	21.62	165.85	Dogny
	MET052	11-2014	rain forest	21.62	165.85	Dogny
	MET053	11-2014	rain forest	21.62	165.85	Dogny
	MET092	04-2014	sclerophyllous forest	22.17	166.79	Pindaï
	MET099	04-2014	sclerophyllous forest	22.17	166.79	Pindaï
*B. vittata*	MET049	11-2014	rain forest	21.62	165.87	Dogny
	MET060	05-2015	rain forest	21.63	165.87	Dogny

## Supplementary Materials

1D and 2D NMR spectra, UV and IR spectra of compounds **23** and **51** are available in the Supplementary Materials.
