# Supplementary material for: Chemotypes and Biomarkers of Seven Species of New Caledonian Liverworts from the Bazzanioideae Subfamily
Source: Molecules. 2018 Jun 5;23(6):1353. doi: 10.3390/molecules23061353 (PMC6100190; doi:10.3390/molecules23061353)
Supplement: Supplementary file 1 [file molecules-23-01353-s001.pdf]

molecules

Article

# Supplementary Materials: Chemotypes and biomarkers of seven species of New Caledonian Liverworts from the Bazzanioideae subfamily

**Benjamin Métoyer, Nicolas Lebouvier, Edouard Hnawia, Gaëtan Herbette, Louis Thouvenot, Yoshinori Asakawa, Mohammed Nour and Phila Raharivelomanana**

molecules

MDPI

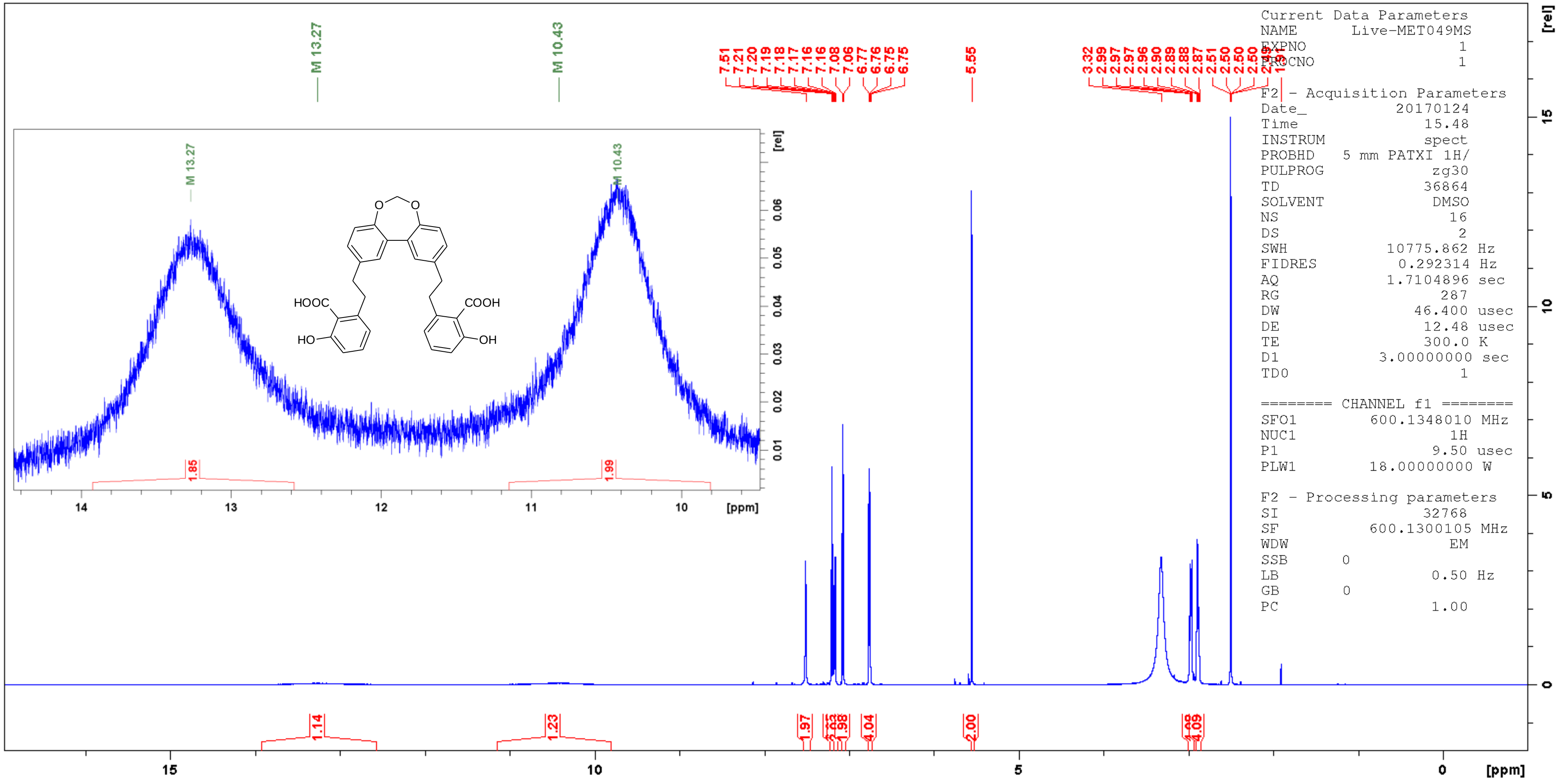

**Figure S1.** $^{1}$H-NMR (600 MHz, DMSO-d6) spectrum of compound **51** (full spectrum).

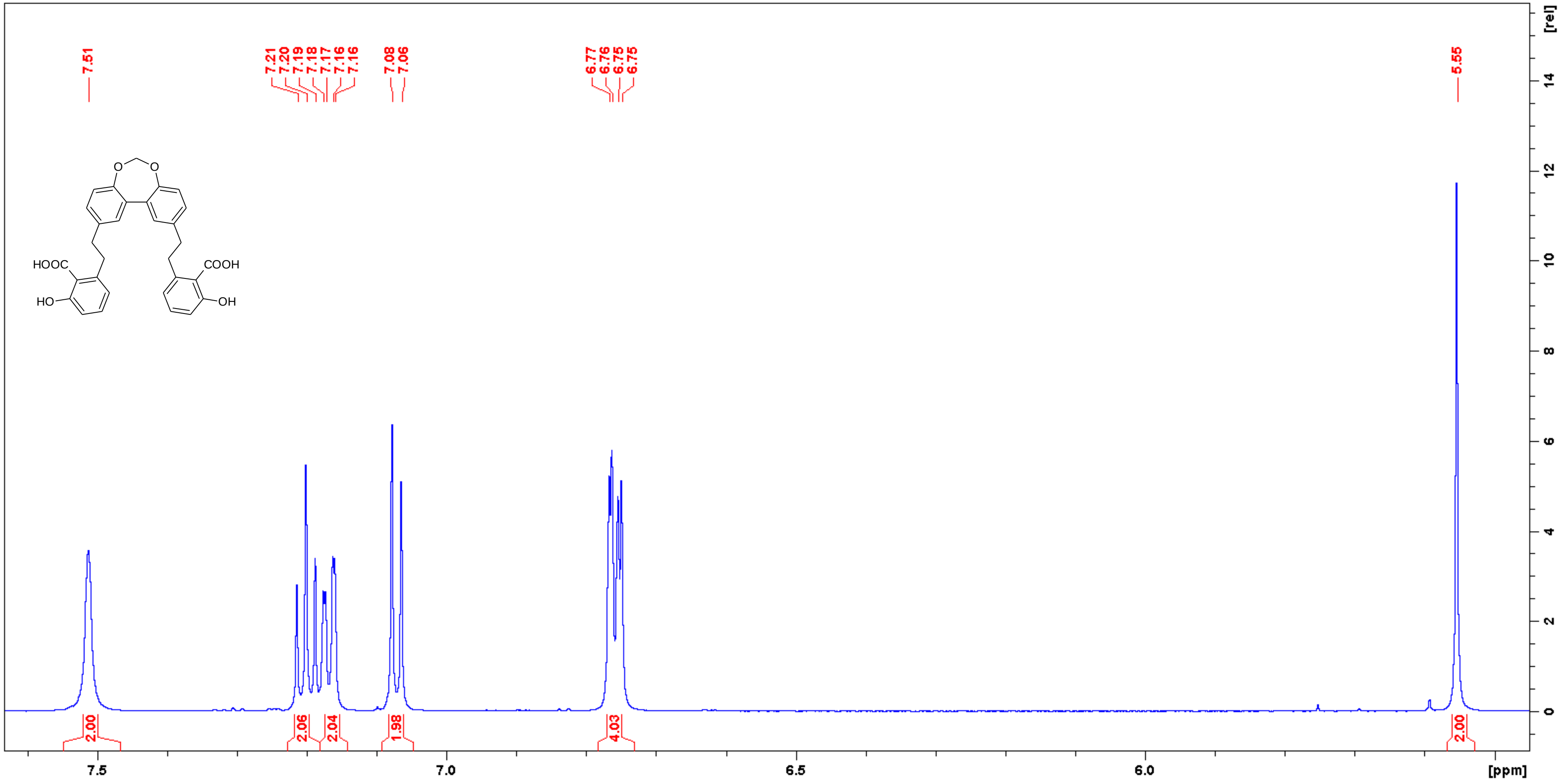


**Figure S2.** $^{1}$H-NMR (600 MHz, DMSO-d6) spectrum of compound **51** (from 5 to 6 ppm).

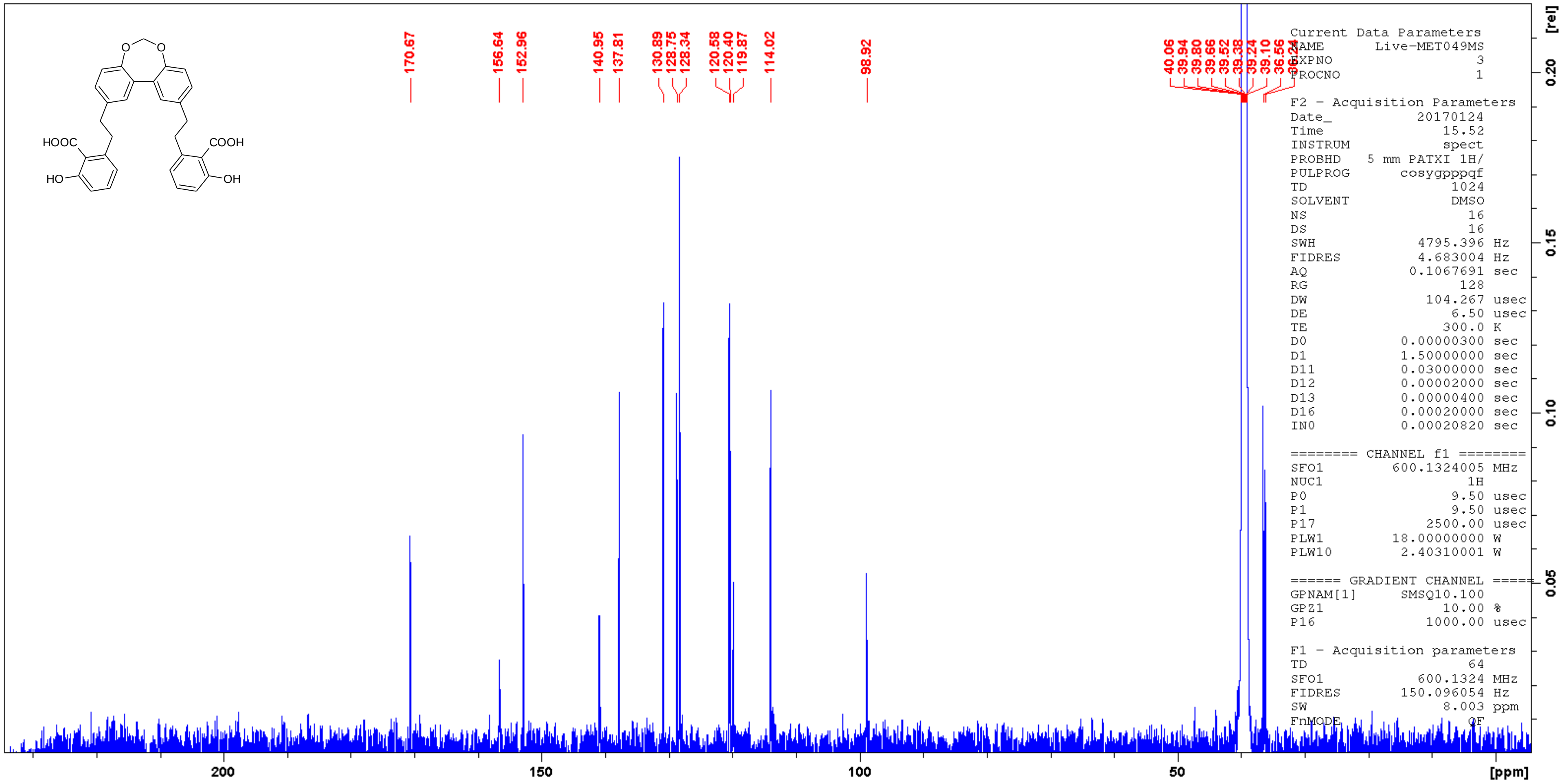


**Figure S3.** $^{13}$C-NMR (150 MHz, DMSO-d6) spectrum of compound **51**.

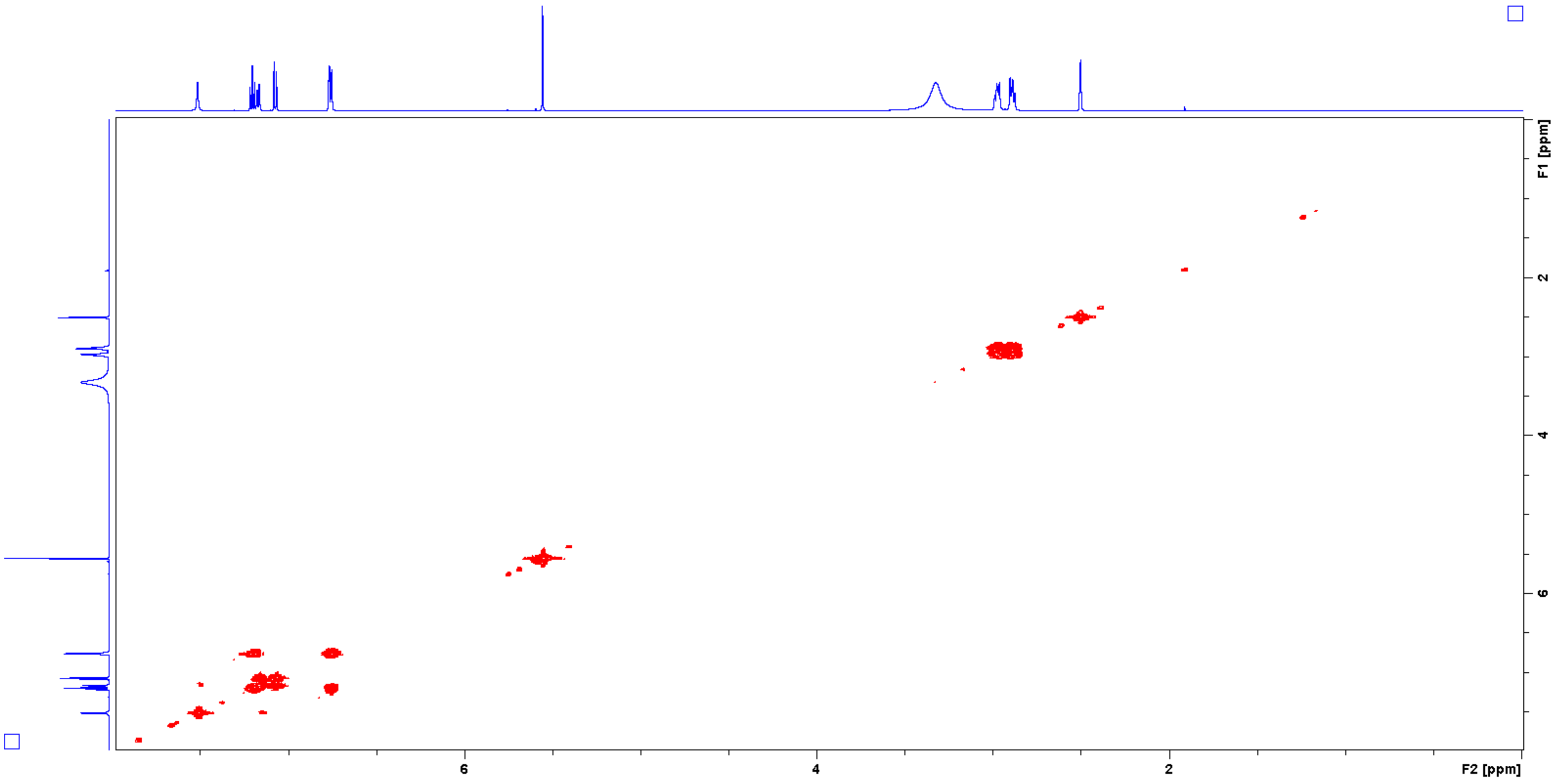


**Figure S4.** $^{1}$H-$^{1}$H COSY NMR spectrum of compound **51**.

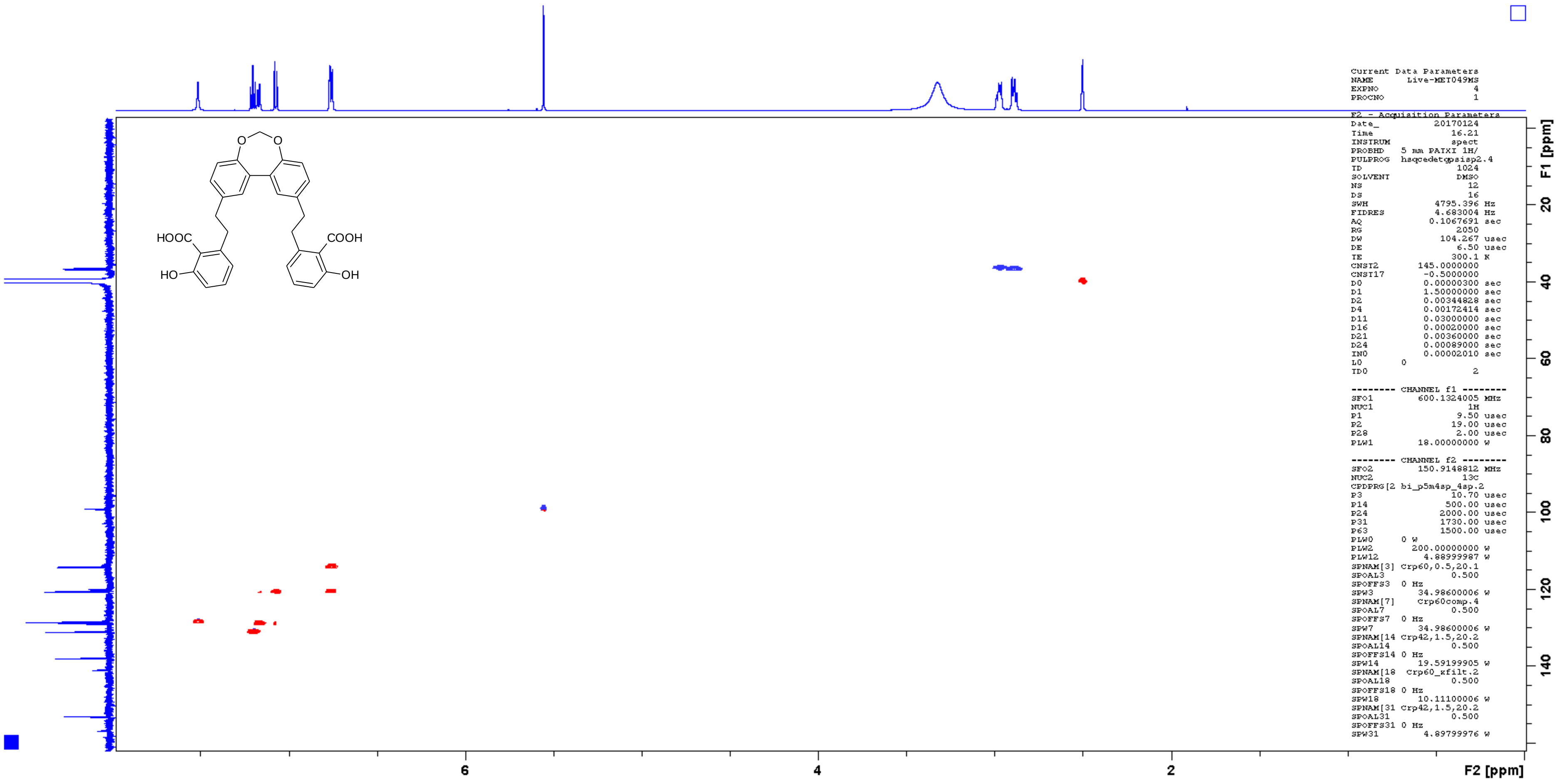


**Figure S5.** $^1H$-$^{13}C$ HSQC NMR spectrum of compound **51**.

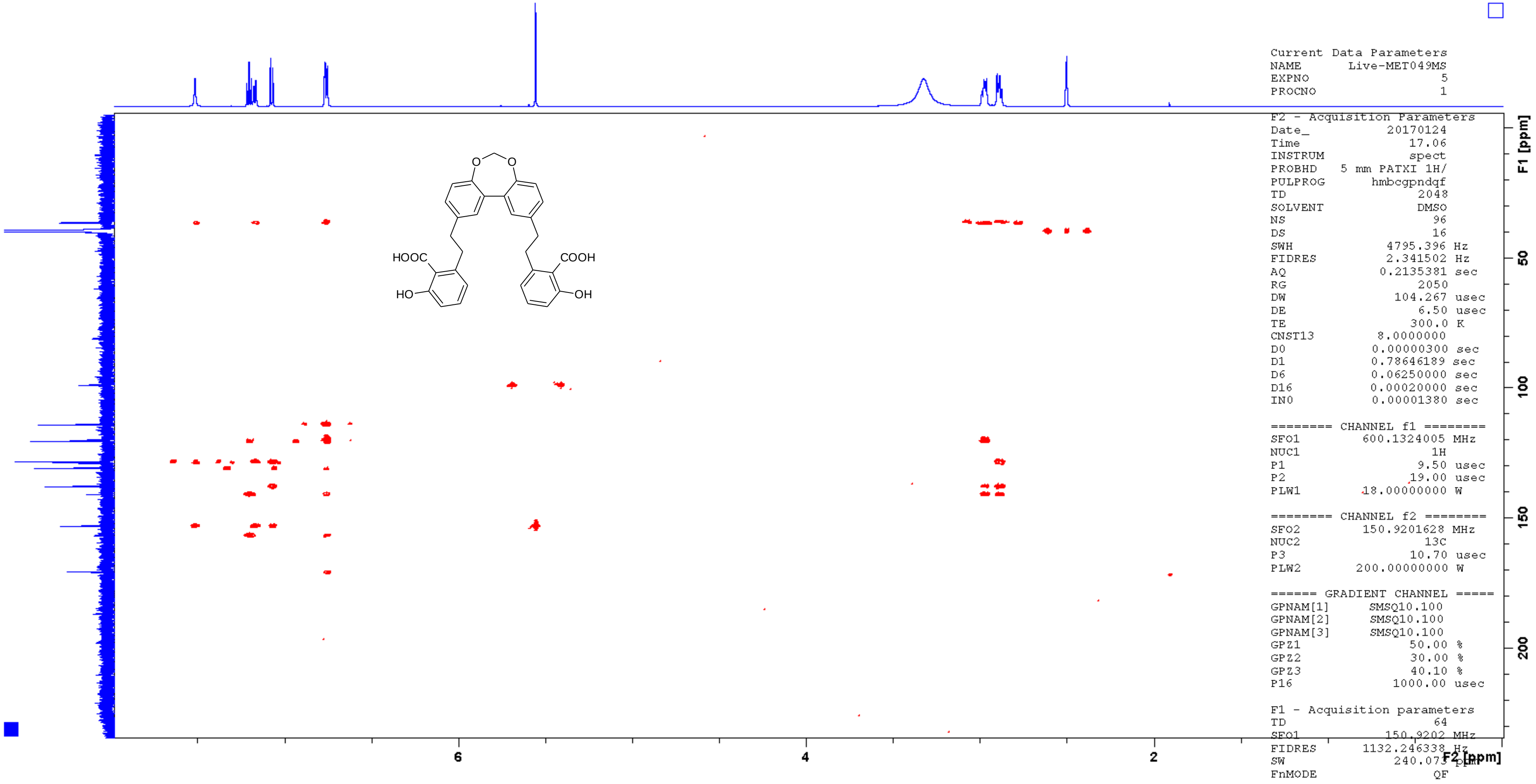


**Figure S6.** $^{1}H$-$^{13}C$ HMBC NMR spectrum of compound **51**.

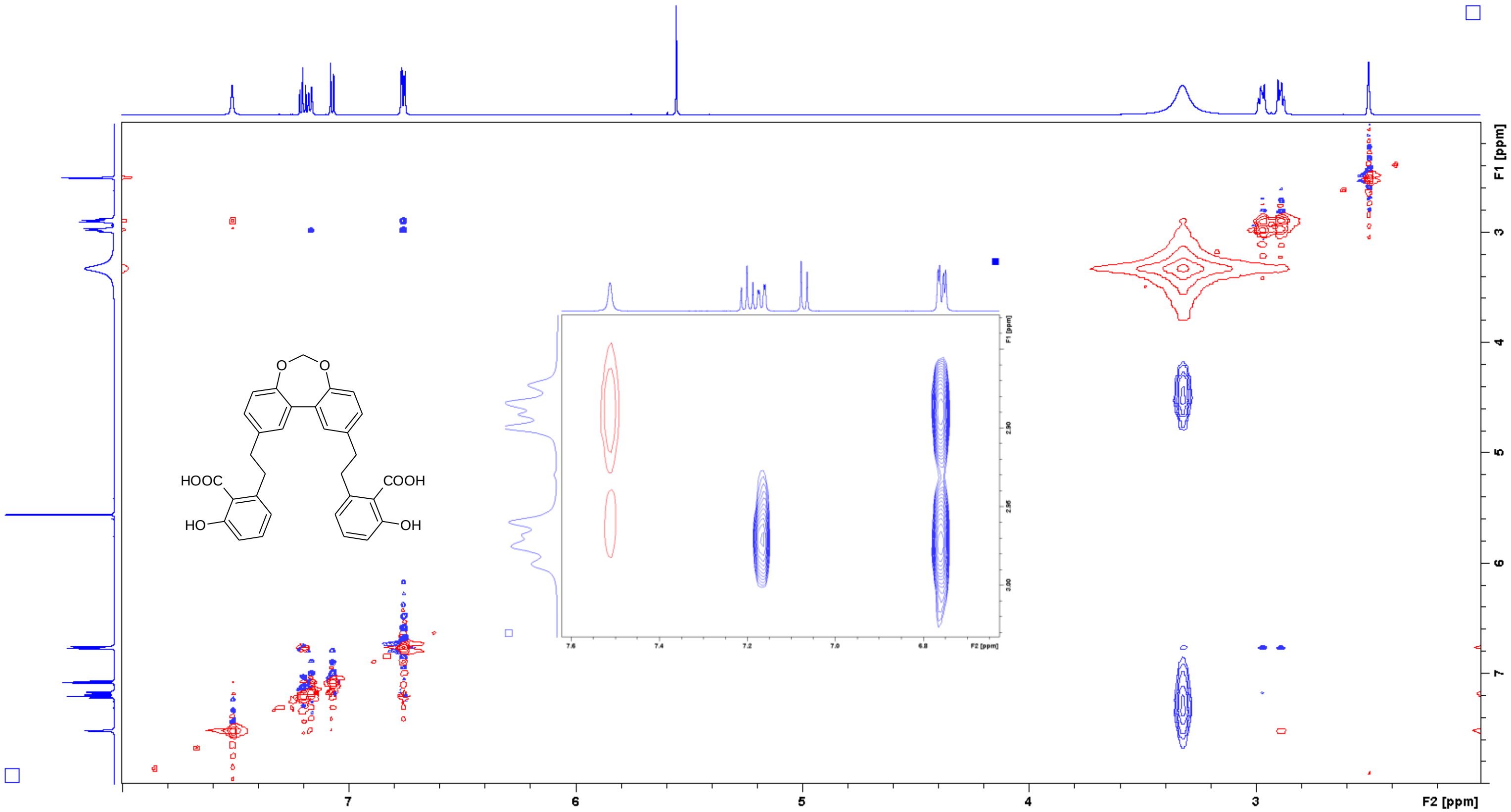


**Figure S7.** $^1$H-$^1$H NOESY NMR spectrum of compound **51**.

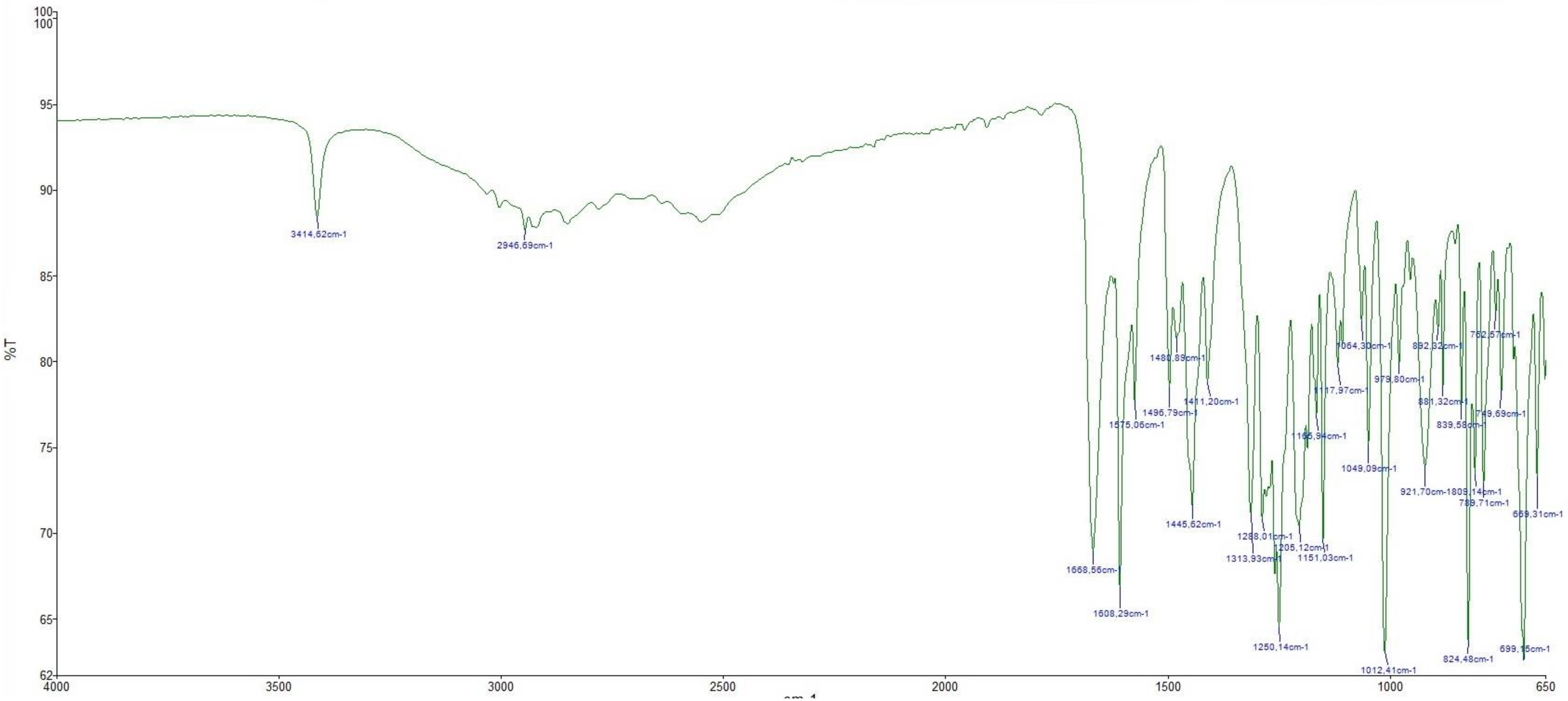


**Figure S8.** Infrared spectrum of compound **51**.

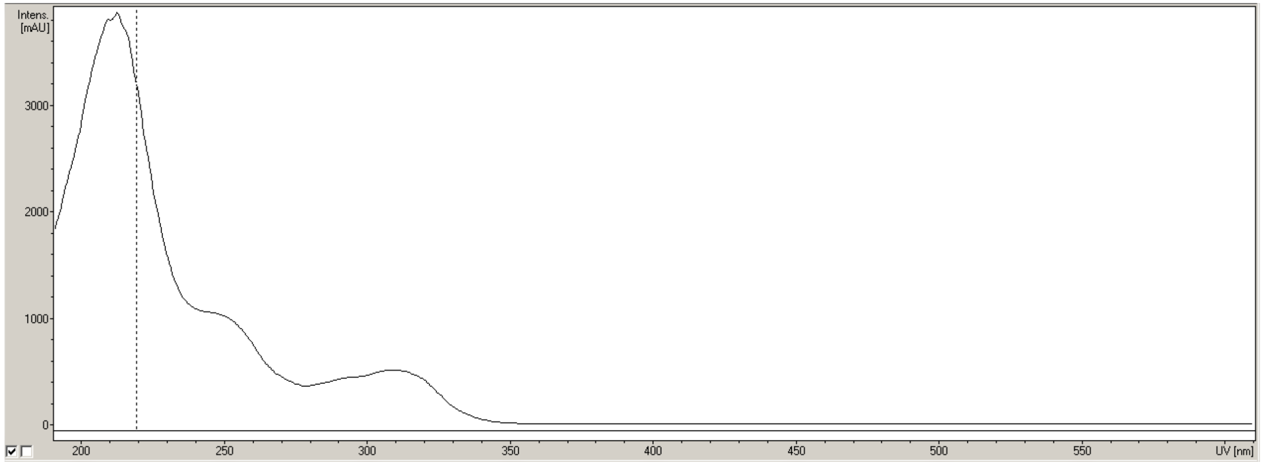


**Figure S9.** Ultraviolet spectrum of compound **51** ($\lambda_1$= 212.50 nm, $\lambda_2$= 247.5 nm, $\lambda_3$= 308.50 nm).

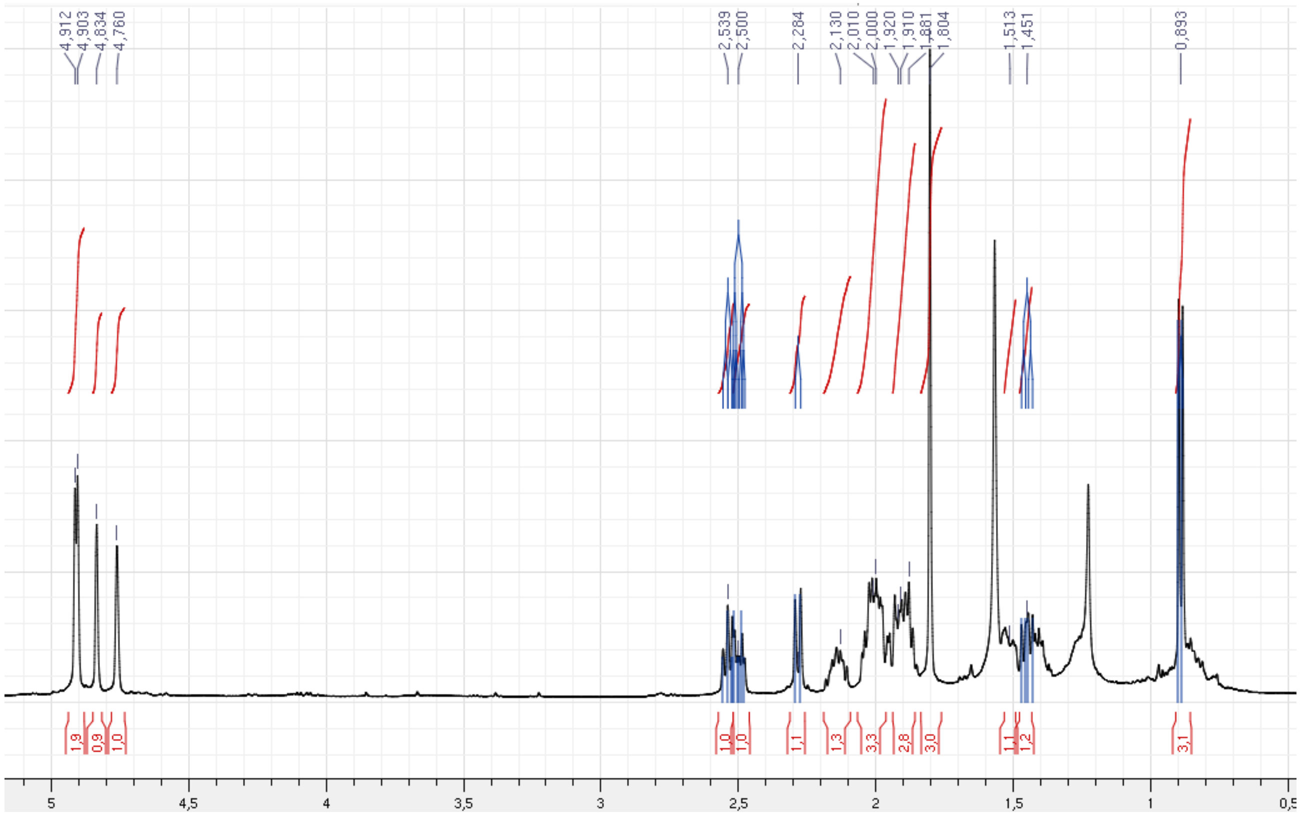


**Figure S10.** $^{1}$H-NMR (500 MHz, $CDCl_3$) spectrum of compound **23**.

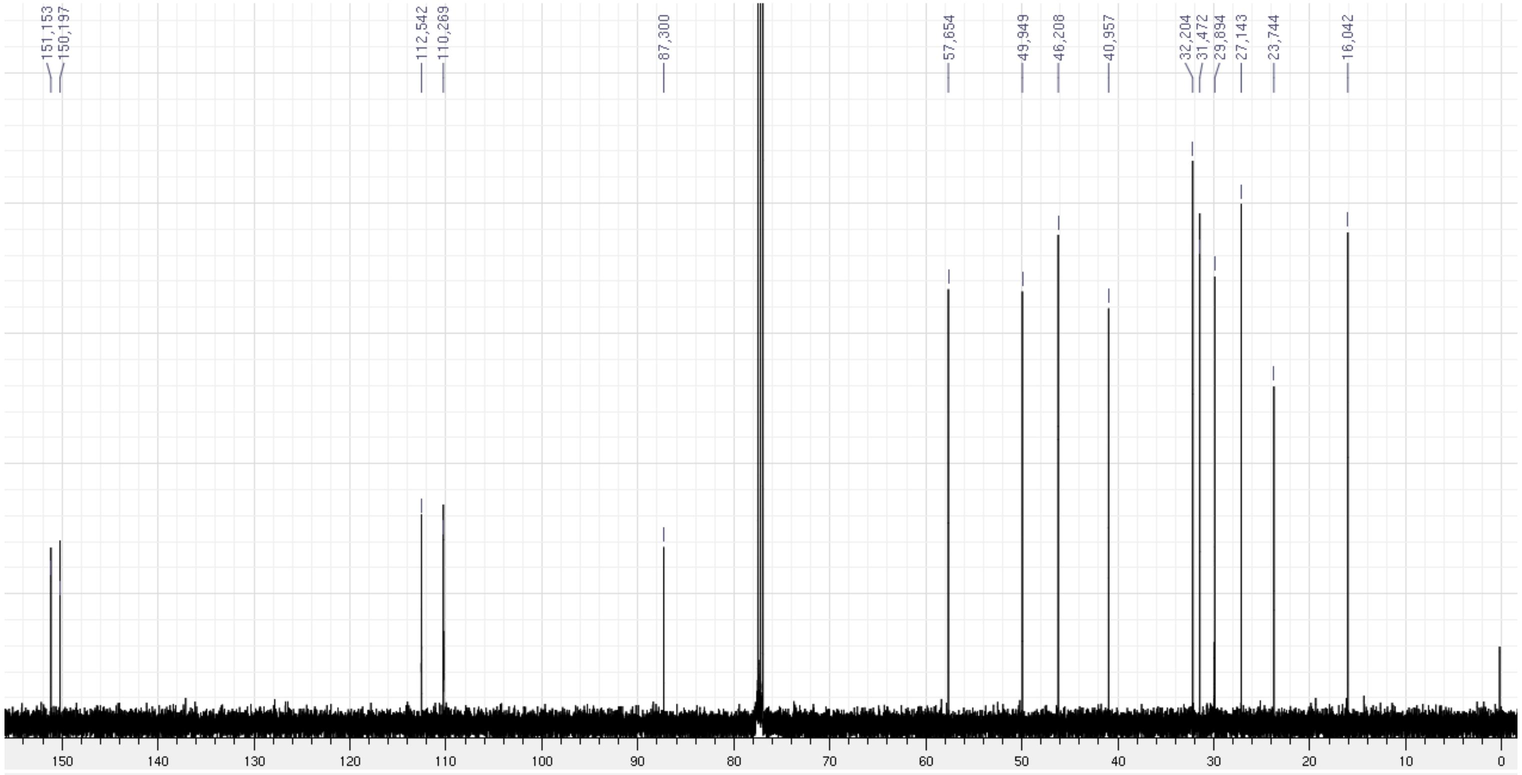


**Figure S11.** $^{13}C$-NMR (125 MHz, $CDCl_3$) spectrum of compound **23**.

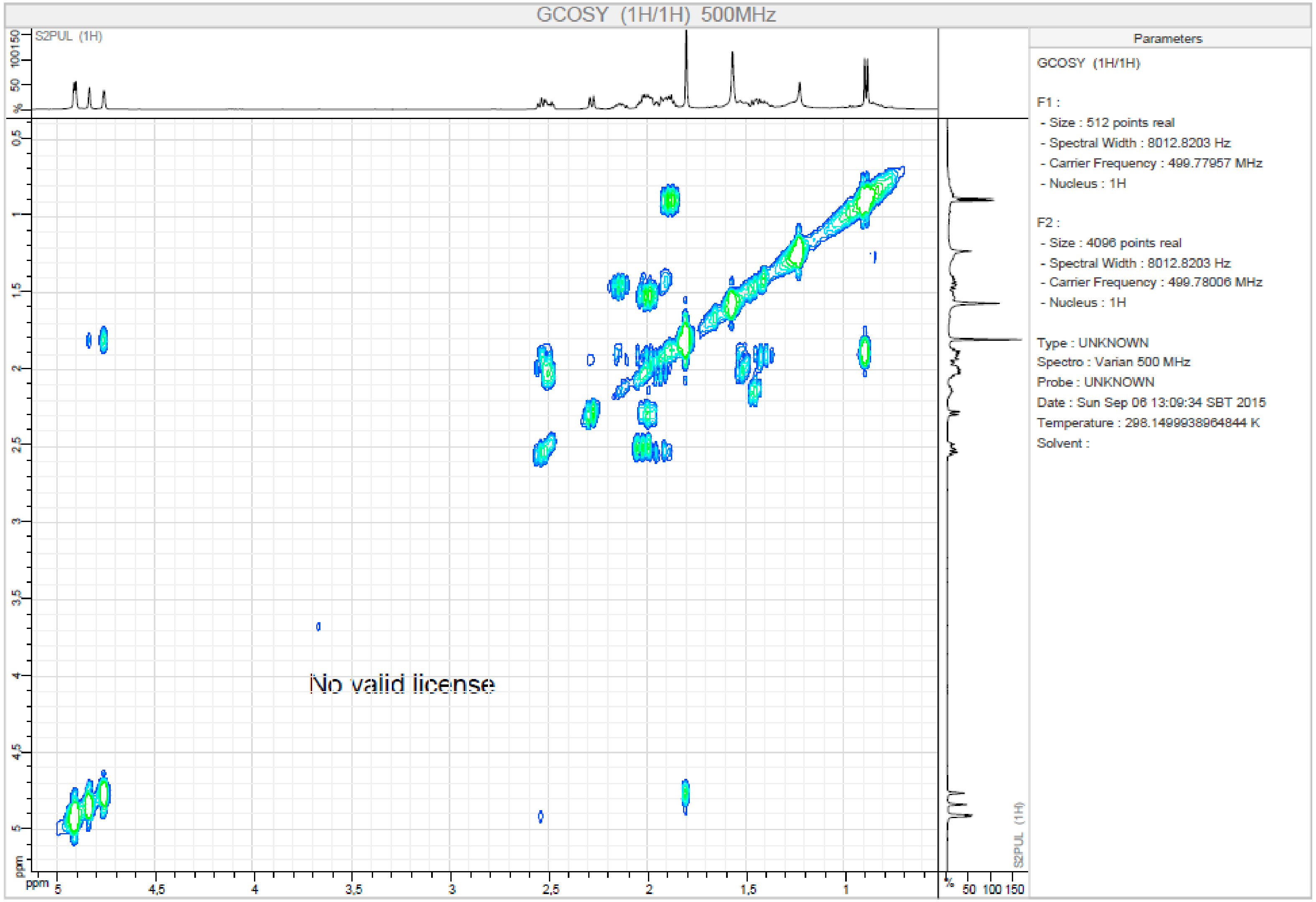


**Figure S12.** $^1H$-$^1H$ COSY NMR spectrum of compound **51**.

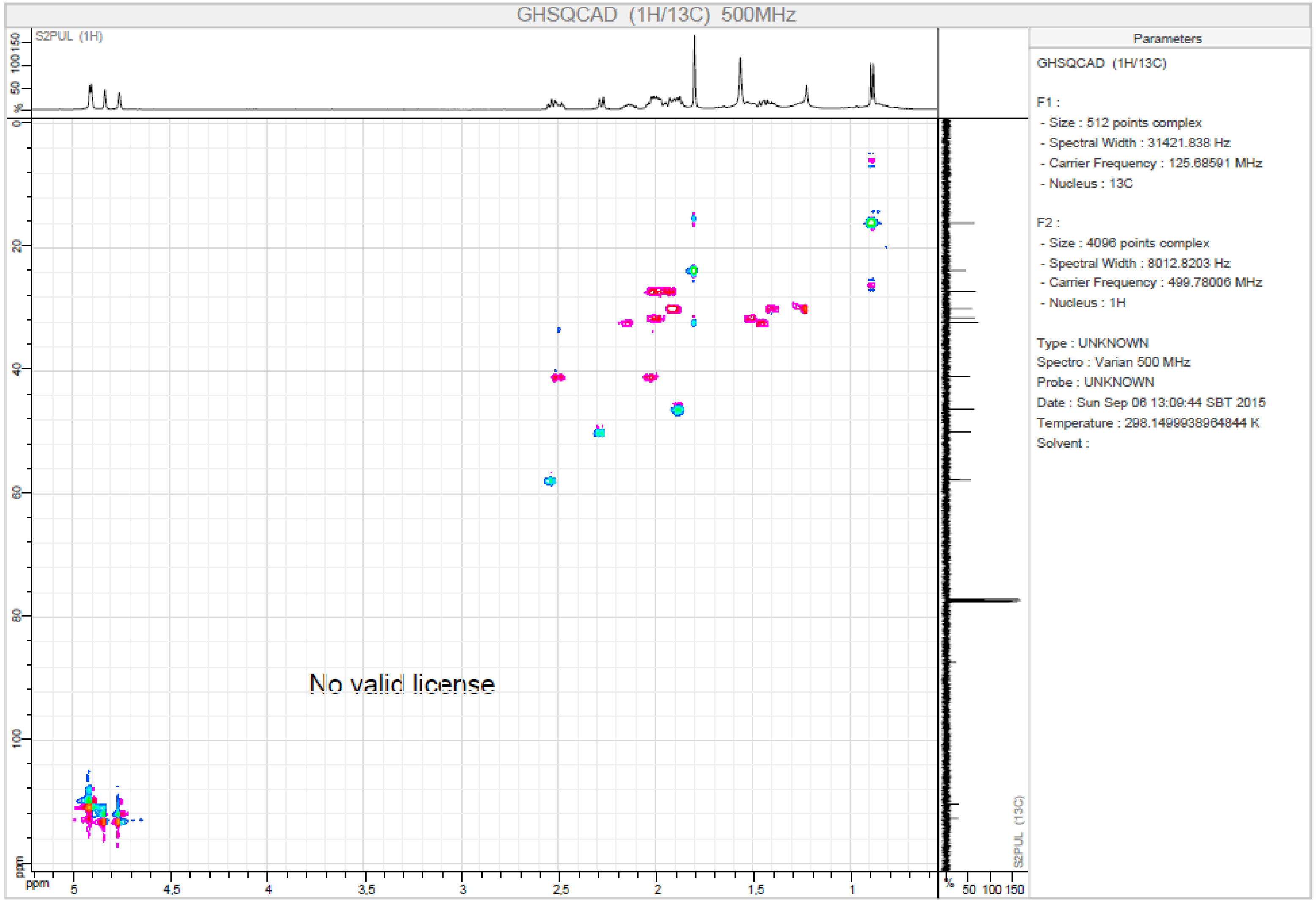


**Figure S13.** $^{1}H$-$^{13}C$ HSQC NMR spectrum of compound **23**.

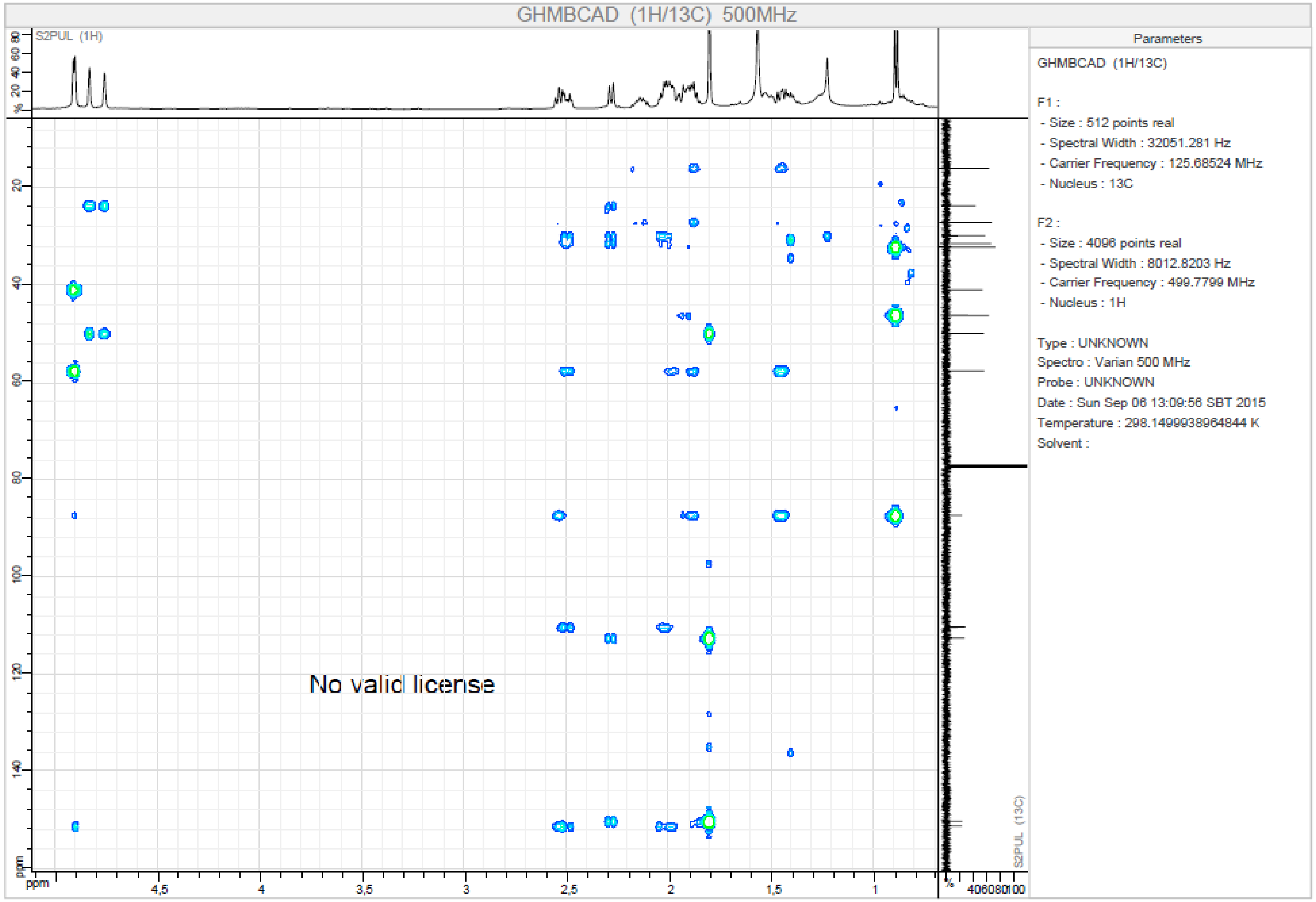


**Figure S14.** $^{1}H$-$^{13}C$ HMBC NMR spectrum of compound **23**.

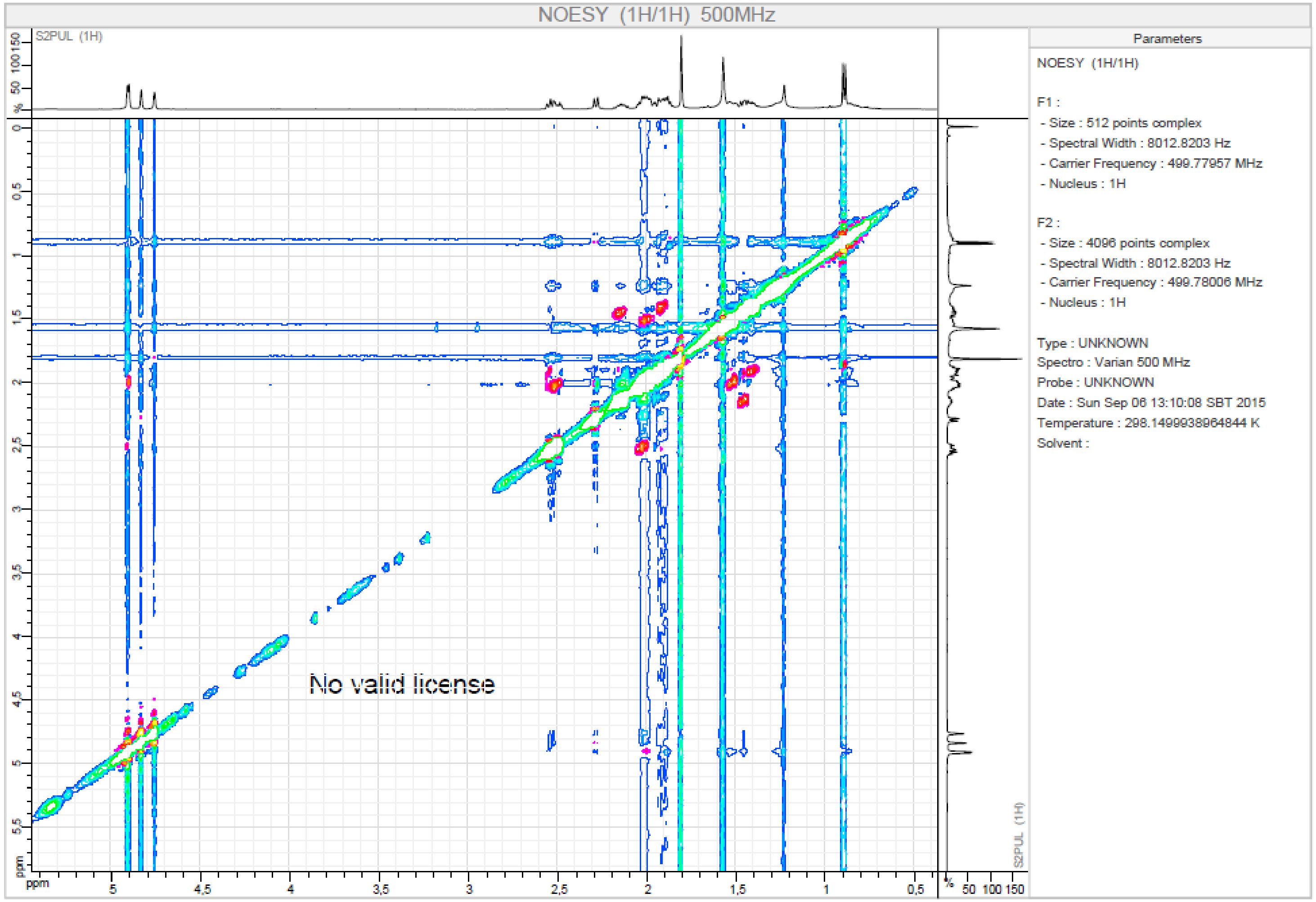


**Figure S15.** $^1$H-$^1$H NOESY NMR spectrum of compound **23**.

molecules

MDPI

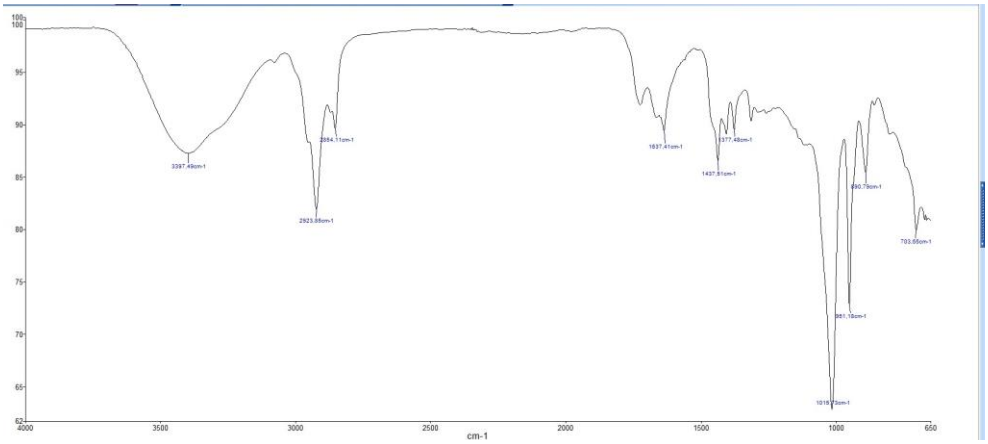


**Figure S16.** Infrared spectrum of compound **23**.

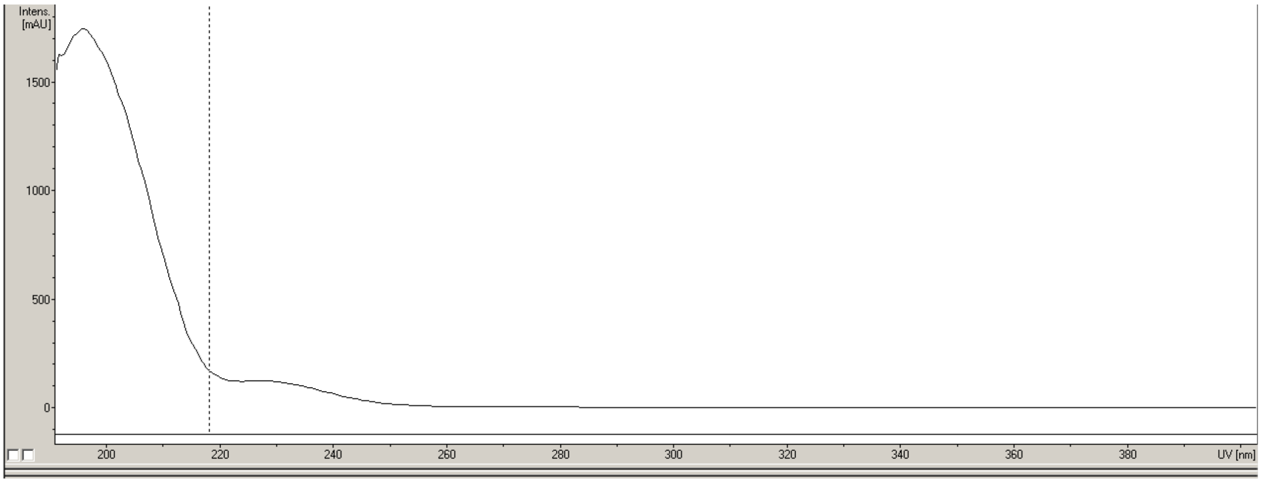


**Figure S17.** Ultraviolet spectrum of compound **23** .
